# Comprehensive bioinformatics analysis identifies metabolic and immune-related diagnostic biomarkers shared between diabetes and COPD using multi-omics and machine learning

**DOI:** 10.3389/fendo.2024.1475958

**Published:** 2025-01-08

**Authors:** Qianqian Liang, Yide Wang, Zheng Li

**Affiliations:** ^1^ Department of Integrated Pulmonology, Fourth Clinical Medical College of Xinjiang Medical University, Urumqi, Xinjiang, China; ^2^ Xinjiang National Clinical Research Base of Traditional Chinese Medicine, The Affiliated Hospital of Xinjiang University of Traditional Chinese Medicine, Urumqi, Xinjiang, China; ^3^ Xinjiang Key Laboratory of Respiratory Disease Research, The Affiliated Hospital of Xinjiang University of Traditional Chinese Medicine, Urumqi, Xinjiang, China; ^4^ Xinjiang Clinical Medical Research Center of Respiratory Obstructive Diseases, The Affiliated Hospital of Xinjiang University of Traditional Chinese Medicine, Urumqi, Xinjiang, China

**Keywords:** diabetes, COPD, machine learning, shared biomarkers, inflammation

## Abstract

**Background:**

Diabetes and chronic obstructive pulmonary disease (COPD) are prominent global health challenges, each imposing significant burdens on affected individuals, healthcare systems, and society. However, the specific molecular mechanisms supporting their interrelationship have not been fully defined.

**Methods:**

We identified the differentially expressed genes (DEGs) of COPD and diabetes from multi-center patient cohorts, respectively. Through cross-analysis, we identified the shared DEGs of COPD and diabetes, and investigated alterations of signaling pathways using Gene Ontology (GO), Kyoto Encyclopedia of Genes and Genomes (KEGG), and gene set enrichment analysis (GSEA). By using weighted gene correlation network analysis (WGCNA), key gene modules for COPD and diabetes were identified, and various machine learning algorithms were employed to identify shared biomarkers. Using xCell, we investigated the relationship between shared biomarkers and immune infiltration in diabetes and COPD. Single-cell sequencing, clinical samples, and animal models were used to confirm the robustness of shared biomarkers.

**Results:**

Cross-analysis identified 186 shared DEGs between diabetes and COPD patients. Functional enrichment results demonstrate that metabolic and immune-related pathways are common features altered in both diabetes and COPD patients. WGCNA identified 526 genes from key gene modules in COPD and diabetes. Multiple machine learning algorithms identified 4 shared biomarkers for COPD and diabetes, including CADPS, EDNRB, THBS4 and TMEM27. Finally, the 4 shared biomarkers were validated in single-cell sequencing data, clinical samples, and animal models, and their expression changes were consistent with the results of bioinformatic analysis.

**Conclusions:**

Through comprehensive bioinformatics analysis, we revealed the potential connection between diabetes and COPD, providing a theoretical basis for exploring the common regulatory genes.

## 1 Introduction

Diabetes is a chronic disease that affects millions of people worldwide, characterized by the body’s inability to regulate blood sugar levels properly (1). Typically, this is due to insufficient insulin secretion or the body’s ineffective use of insulin, the hormone responsible for regulating blood sugar. The global prevalence of diabetes has been steadily rising, posing significant challenges to public health systems and the overall well-being of affected individuals (2). Current research efforts are primarily aimed at understanding the impact of genetic, environmental, and lifestyle factors on diabetes. Furthermore, investigations into the pathogenesis of the disease, related complications, and therapeutic interventions are underway. Despite significant progress in these areas, the exact mechanisms of diabetes and its various subtypes remain elusive, requiring further exploration and innovative approaches (3).

Chronic obstructive pulmonary disease (COPD) is a group of progressive lung diseases, including emphysema and chronic bronchitis, characterized by obstructed airflow in the lungs (4, 5). The global burden of chronic obstructive pulmonary disease has been on the rise, affecting more and more individuals with debilitating symptoms such as breathlessness, chronic cough, and sputum production (6, 7).

Research in the field of COPD is diverse, encompassing investigations into the etiology, risk factors, diagnostic methods, and treatment strategies of the disease (8, 9). Despite significant progress in understanding the pathophysiology of COPD, including the roles of oxidative stress, inflammation, and genetic susceptibility, a comprehensive understanding of the molecular basis and precise biomarkers related to disease progression and severity has not been fully elucidated (10, 11).

Emerging evidence suggests a significant overlap between these diseases, beyond their traditional classifications as metabolic and pulmonary disorders. Epidemiological studies have demonstrated a higher prevalence of COPD in diabetic patients and an increased risk of diabetes in COPD patients. For instance, a recent prospective cohort study from the UK Biobank reported that prediabetic individuals and diabetic patients had an 18% and 35% higher risk of developing COPD, respectively (12). This bidirectional relationship underscores the need to explore shared biological pathways.

Both diabetes and COPD are characterized by systemic inflammation, metabolic dysregulation, and immune system disturbances. For example, studies have shown that inflammatory mediators, such as interleukins and cytokines, are elevated in both conditions, contributing to disease progression (13). Additionally, oxidative stress and mitochondrial dysfunction are common features in both diseases, suggesting overlapping pathophysiological mechanisms (14). These shared pathways highlight the potential for identifying common biomarkers that can improve early diagnosis and therapeutic strategies.

Despite significant research into the independent mechanisms of diabetes and COPD, the molecular connections between these two diseases remain poorly understood. Recent advances in multi-omics technologies and machine learning offer unprecedented opportunities to uncover shared biomarkers. By integrating genomics, transcriptomics, and proteomics data, this study aims to identify molecular features that transcend traditional disease boundaries. Such biomarkers not only have the potential to elucidate the complex interrelationship between diabetes and COPD but also provide insights into broader systemic diseases with shared inflammatory and metabolic underpinnings.

In summary, diabetes and COPD are prominent global health challenges, each imposing significant burdens on affected individuals, healthcare systems, and society. While extensive research has deepened our understanding of these diseases, critical knowledge gaps still exist, particularly in their common molecular basis and potential interactions. To address this gap, this study leverages publicly available, high-quality datasets from the Gene Expression Omnibus (GEO), a comprehensive repository for gene expression data. The datasets utilized include transcriptomic profiles from lung tissues of COPD patients (GSE38974, GSE69818, GSE76925) and pancreatic islets tissues from diabetes patients (GSE41762, GSE50398). Our research has revealed shared biomarkers through multi-omics analysis, which is a crucial step in elucidating the complex relationship between diabetes and COPD. The significance of our findings goes beyond these individual conditions, providing insights into broader disease mechanisms and paving the way for novel diagnostic and therapeutic strategies with potential interdisciplinary applications.

## 2 Materials and methods

### 2.1 Microarray data acquisition and preprocessing

Microarray data and clinical information for lung tissues of COPD patients (GSE38974, GSE69818, GSE76925) and pancreatic islets tissues of diabetes patients (GSE41762, GSE50398) were obtained from the GEO database. Data preprocessing, including batch effect removal and gene ID conversion, was performed using R packages (GEOquery, tinyarray, AnnoProbe, limma), resulting in integrated datasets for COPD and diabetes. Ultimately, the integrated multi-center COPD microarray data consisted of 253 samples, including 49 normal samples and 204 COPD samples. Integrated multi-center diabetes microarray data consisted of 166 samples, including 57 normal samples and 109 diabetes samples.

### 2.2 Differential gene expression analysis

**Figure 1:**
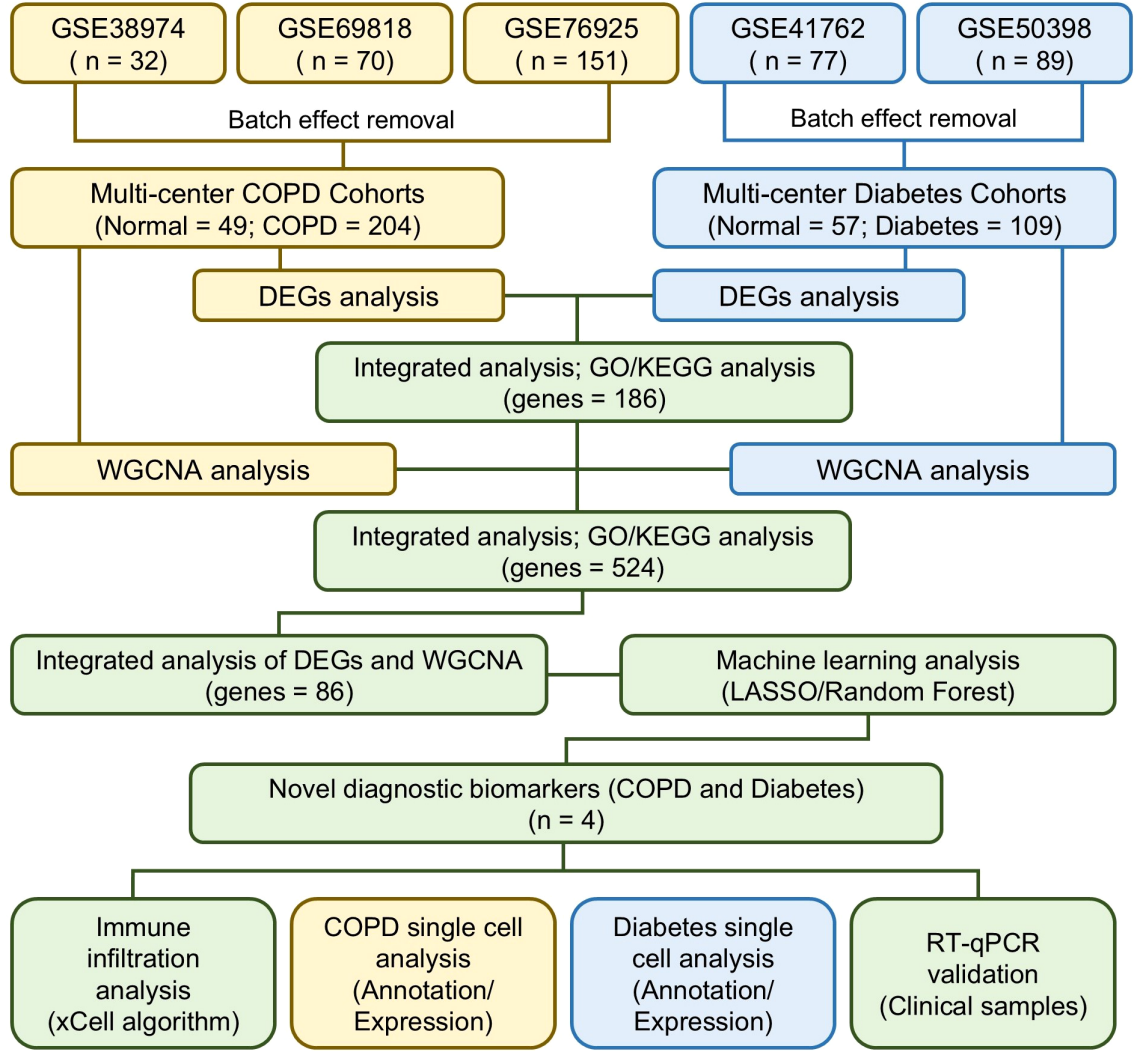
Flow chart of this study design.

The R packages “tinyarray”, “AnnoProbe”, and “limma” were used for background correction, data normalization, and gene symbol conversion in the COPD and diabetes microarray datasets. Differentially expressed genes (DEGs) were identified using limma with default parameters, defining genes with fold change >1 and P<0.05 as upregulated, and fold change <1 and P<0.05 as downregulated. The DEGs were visualized as volcano plots and heatmaps using ggplot2 and pheatmap. Shared DEGs between COPD and diabetes cohorts were identified through cross-analysis and visualized in a Venn diagram. The study design is presented in the flow chart (**Figure 1**).

### 2.3 Weighted gene correlation network analysis

WGCNA is a frequently employed method for analyzing gene co-expression networks, aiming to discover correlations and patterns among genes. In brief, we filtered 5000 highly variable genes from multi-center COPD and diabetes datasets, and conducted data quality control using “goodSamplesGenes” function of R package “WGCNA”. Subsequently, we performed unsupervised clustering on the input data using default parameters and constructed a scale-free co-expression gene network. Furthermore, we established an appropriate soft threshold power and obtained different module eigengenes (ME) based on the first principal component of module expression to evaluate the correlation between modules and traits. Ultimately, we filtered modules significantly associated with traits based on *P* < 0.05 and extracted genes contained in each module as key genes for the disease.

### 2.4 Gene functional enrichment analysis

To explore the molecular functions associated with the target gene set, we initially used the R package “clusterProfiler” to convert gene symbols. Subsequently, using the default parameters of the R package “clusterprofiler”, we conducted enrichment analysis on functional gene sets from the Gene Ontology (GO) database (https://www.geneontology.org/) and the Kyoto Encyclopedia of Genes and Genomes (KEGG) database (https://www.kegg.jp/). The enrichment results were visualized using the R packages “ggplot2” and “GOplot”. The gene set enrichment analysis (GSEA) was performed using the R packages “GSEAbase” and “clusterProfiler”, with the DEGs from multi-center COPD and diabetes cohorts sorted by fold change. Pathways with *P* < 0.05 were identified as significantly altered and visualized using the R package “GseaVis”.

### 2.5 Machine learning algorithms

In this study, the least absolute shrinkage and selection operator (LASSO) algorithm and random forest algorithm were employed to identify shared biomarkers for COPD and diabetes, because they are well-suited for high-dimensional datasets with numerous predictors, such as the gene expression data in this study. LASSO regression effectively performs feature selection by imposing L1 regularization, thus reducing model complexity and mitigating overfitting (15). Meanwhile, the Random Forest algorithm provides robust performance in handling nonlinear relationships and assessing feature importance (16). The combination of these two methods ensures that our biomarker identification process is both rigorous and reliable. These algorithms were chosen to complement each other, reducing the bias that may arise from relying on a single model. Alternative approaches, such as support vector machines or deep learning, were not employed due to their computational complexity and the study’s focus on feature selection rather than prediction. In brief, for the multi-center COPD and diabetes cohorts, we randomly split all samples into training and testing sets at a 7:3 ratio. Using the default parameters of the R packages “glmnet” and “randomForest”, we constructed predictive models for COPD and diabetes based on LASSO regression analysis and random forest algorithm on the training set. Subsequently, we utilized the R package “ROCR” to conduct receiver operating characteristic (ROC) analysis to assess the predictive capability of the model. To narrow down the range of candidate shared biomarkers, we conducted a cross-analysis of key genes in different predictive models and presented the analysis results using a Venn diagram.

### 2.6 Immune infiltration and correlation analysis

The infiltration proportion of immune cells was calculated using the “xCell” R package. In brief, following normalization of the gene expression matrix from multi-center COPD or diabetes cohorts, the infiltration proportions of 64 types of immune cells were calculated using the default parameters of “xCell”. Afterwards, the “ggplot2” R package was utilized to visualize the proportions of the immune cells of interest, and the significance of the differences between the control and disease groups was assessed using the Wilcoxon test, with *P* < 0.05 considered as having significant differences. The Spearman correlation analysis between shared biomarkers and immune cell infiltration proportions was conducted using the “ggcor” R package, and visualized using “pheatmap” R package, with *P* < 0.05 identified as indicating significant correlation.

### 2.7 Single-cell RNA-seq data acquisition and preprocessing

Single-cell RNA-seq data of COPD and diabetes patients were downloaded from the GEO database, including GSE173896 (17) and GSE244515 (18). Quality control of the single-cell RNA-seq data was performed using the “Seurat” R package, and cells with nFeature > 500 were chosen for further analysis. Following this, the R package “harmony” was employed to eliminate batch effects between samples, and the “Seurat” R package was used for uniform manifold approximation and projection (UMAP) dimensionality reduction and cell clustering. Leveraging extensively validated marker genes, we annotated different cell clusters, and identified all differential genes using the “FindAllMarkers” function. The visualization of shared biomarkers was accomplished using “ggplot2” and the “Seurat” R packages.

### 2.8 Collection and processing of clinical samples

Serum samples were collected from a total of 120 individuals, including 40 healthy controls, 40 patients with COPD, and 40 patients with diabetes. The samples were obtained following informed consent and in accordance with the ethical guidelines approved by the institutional review board (IRB). Total RNA was extracted from the serum samples using the FastPure Cell/Tissue Total RNA Isolation Kit (Vazyme), following the manufacturer’s protocol. Quantitative PCR (qPCR) was performed to quantify the expression levels of the identified biomarkers.

### 2.9 Construction of COPD and diabetic mouse models

Twenty male C57BL/6 mice were randomly assigned to control and COPD groups (n=10 per group). The COPD model was induced by exposing mice to cigarette smoke and intraperitoneal injections of cigarette smoke extract (CSE, 0.3 mL/20 g) on days 1, 12, and 23, repeated for 28 days. Control mice received PBS injections (0.3 mL/20 g). Additionally, ten 7-week-old SPF male BKS-db/db mice (39.34 ± 0.34 g) and ten BKS-db/+ mice (28.80 ± 0.46 g) were used as diabetic and control groups, respectively. All animals were housed in the Xinjiang Medical University animal facility. Lung and pancreas tissues were fixed in 4% paraformaldehyde for immunofluorescence or snap-frozen in liquid nitrogen for Western blot analysis. Primary antibodies included TMEM27 (Abcam), THBS4 (Abcam), EDNRB (Proteintech Group), and CAPS1 (Proteintech Group).

### 2.10 Statistical analysis

The statistical analysis was performed using R (4.1.1) software and GraphPad Prism (9.0.2) software. Quantitative data is presented as mean ± standard deviation (mean ± sd). Between-group differences were calculated using unpaired Student’s t-test or one-way ANOVA, with *P* < 0.05 considered statistically significant.

## 3 Results

### 3.1 Dysregulation of immune and metabolic pathways mediates the co-progression of diabetes and COPD

**Figure 2:**
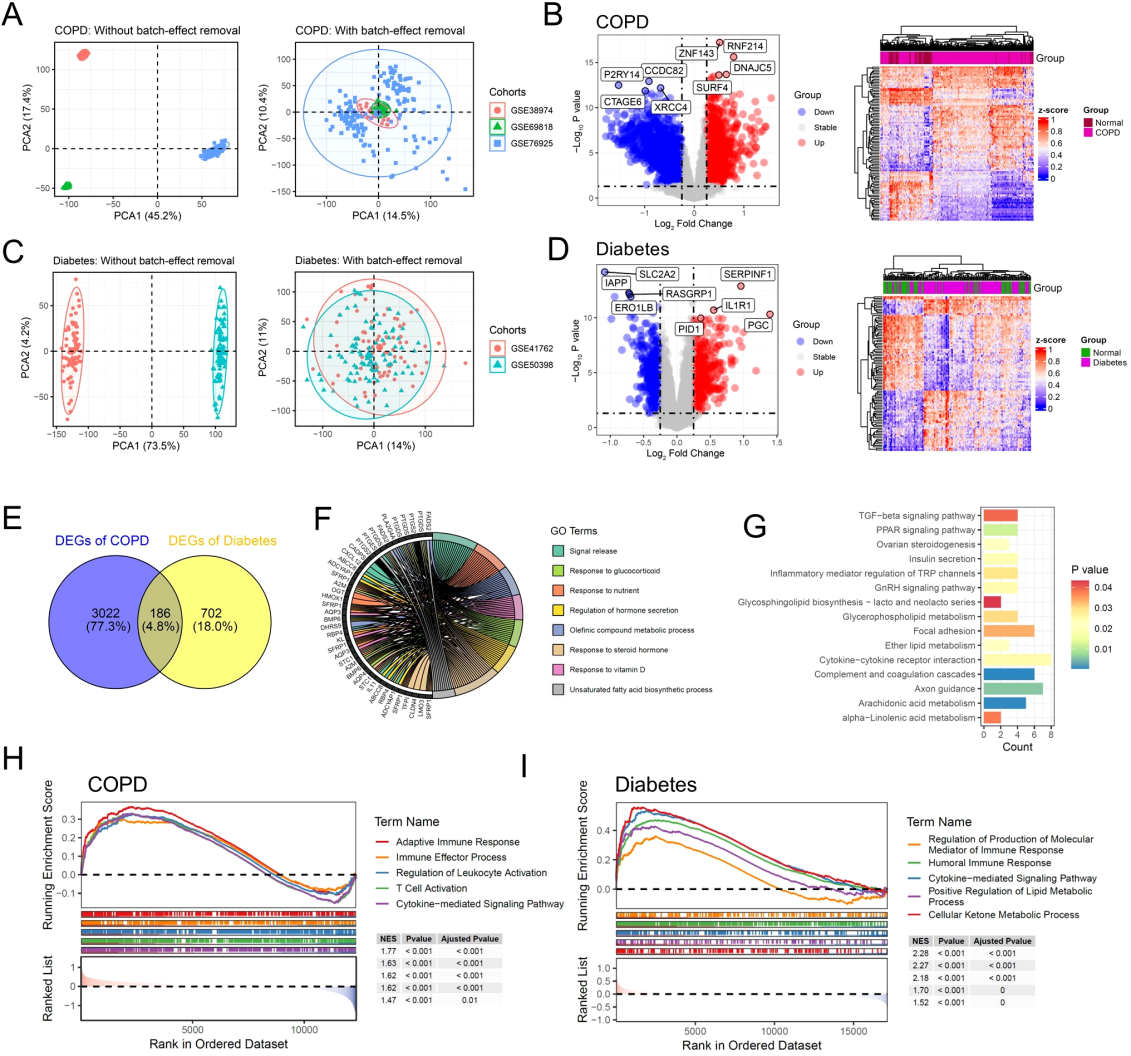
Dysregulation of immune and metabolic pathways mediates the co-progression of diabetes and COPD. **(A)** Scatter plot illustrating the PCA analysis results of the multi-center COPD cohort before and after batch effect removal. **(B)** Volcano plot and heatmap displaying the DEGs of multi-center COPD patient cohort. **(C)** Scatter plot illustrating the PCA analysis results of the multi-center diabetes cohort before and after batch effect removal. **(D)** Volcano plot and heatmap displaying the DEGs of multi-center diabetes cohort. **(E)** Venn diagram illustrating the shared DEGs between the COPD and Diabetes cohorts. **(F)** Chord diagram displaying the enriched GO signaling pathways of the common DEGs. **(G)** Bar plot displaying the enriched KEGG signaling pathways of the common DEGs. **(H)** GSEA results showing the signaling pathways significantly activated in COPD patients. **(I)** GSEA results showing the signaling pathways significantly activated in diabetic patients.

To investigate the significant molecular mechanisms in the pathogenesis of COPD, we integrated microarray data from GSE38974, GSE69818, and GSE76925. After data cleaning and integration, we obtained multi-center COPD microarray data from three projects with batch effects removed, including 49 normal samples and 204 COPD samples (**Figure 2A**). The differential expression analysis results indicated significant alterations in the transcriptome between the COPD group and the normal group (**Figure 2B**). Compared to the normal group, 3208 differentially expressed genes (DEGs) were identified in the COPD group, including 1932 upregulated DEGs and 1276 downregulated DEGs (**Figure 2B**). It was worth noting that, among the significantly upregulated DEGs, ZNF143 had been reported to be associated with the occurrence of COPD and immune infiltration (19). In addition, SURF4 had been confirmed to be involved in autoimmune lung diseases, which indirectly demonstrates the robustness of the analysis in this study (20). To explore the molecular mechanisms of diabetes progression, we re-integrated and obtained multi-center diabetes microarray data, including 57 normal samples and 109 diabetes samples. The principal component analysis (PCA) results indicated that the microarray data of diabetes obtained from GSE41762 and GSE50398 had removed the batch effects (**Figure 2C**). Compared to the normal group, the expression levels of 393 genes were significantly upregulated in the diabetes group, while 495 genes were significantly downregulated (**Figure 2D**). Interestingly, several DEGs identified in this study had been reported to be associated with the occurrence and progression of diabetes, including SERPINF1, PID1, IL1R1 and PGC (21–24).

To further investigate the shared pathogenic mechanisms of COPD and diabetes, we conducted a cross-analysis between 3208 DEGs of COPD and 888 DEGs of diabetes. The results showed that 186 shared DEGs were identified, which were significantly altered in both COPD and diabetes (**Figure 2E**). We performed gene function enrichment analysis to investigate the molecular functions and signaling pathways in which the 168 shared DEGs were involved. Consistent with previous reports, the gene ontology (GO) results indicated significant enrichment of pathways related to metabolism and immunity, including signal release, regulation of hormone secretion and response to steroid hormone (**Figure 2F**) (14, 25). KEGG enrichment analysis demonstrated similar results, with significant enrichment of metabolic and inflammatory pathways, including inflammatory mediator regulation of TRP channels and arachidonic acid metabolism (**Figure 2G**). We further performed gene set enrichment analysis (GSEA) to investigate the activation of inflammatory and metabolic pathways. The results showed that compared to the control group, inflammatory and metabolic pathways were significantly activated in patients with diabetes or COPD (**Figures 2H, I**).

### 3.2 Identification of key gene expression patterns of COPD using WGCNA algorithm

**Figure 3:**
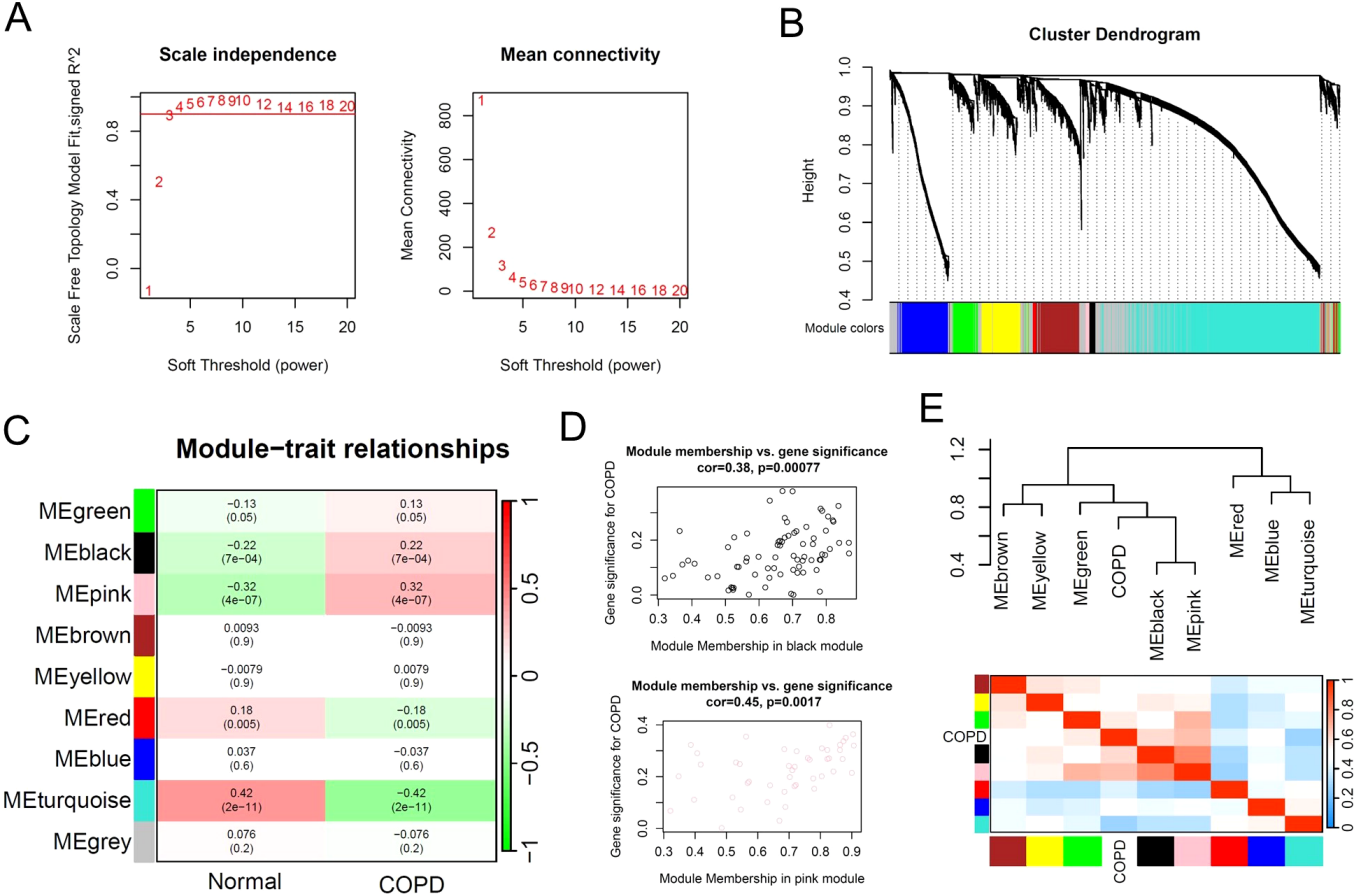
Identification of key gene expression patterns of COPD using WGCNA algorithm. **(A)** Scale-free topology model identifying the best β value, and β = 3 was chosen as the soft threshold based on the average connectivity and scale independence. **(B)** Cluster dendrogram shows the characteristic genes of different modules. **(C)** Heatmap displays the correlation of different gene modules with COPD. **(D)** Scatter plot displaying the correlation between representative gene modules and COPD. **(E)** Hierarchical clustering dendrogram and heatmap illustrating the Pearson correlation between COPD and various gene modules.

To identify the key gene modules in COPD patients, we performed weighted gene correlation network analysis (WGCNA) based on the multi-center COPD microarray data. The results showed that during the construction of the gene co-expression network, when the soft threshold (power) was set to 3, the scale-free topology model fit reached 0.9 (**Figure 3A**). Subsequently, we set the MEDissThres parameter to 0.3 and used the dynamic tree cut algorithm to identify similar gene expression modules. The results showed that based on the similarity of gene expression, 9 modules were ultimately identified for subsequent analysis (**Figure 3B**). With the default parameters, we calculated the correlation between gene expression modules and phenotypes. Based on the correlation coefficients and p-values, we selected the black, pink, and turquoise modules as key modules, comprising a total of 2537 genes (**Figures 3C, D**). The dendrogram and heatmap illustrated the correlation between different gene expression modules and the COPD phenotype, with the black and pink modules showing a significant positive correlation with COPD, while the turquoise module demonstrated a significant negative correlation with COPD (**Figure 3E**).

### 3.3 Identification of key gene expression patterns of diabetes using WGCNA algorithm

**Figure 4:**
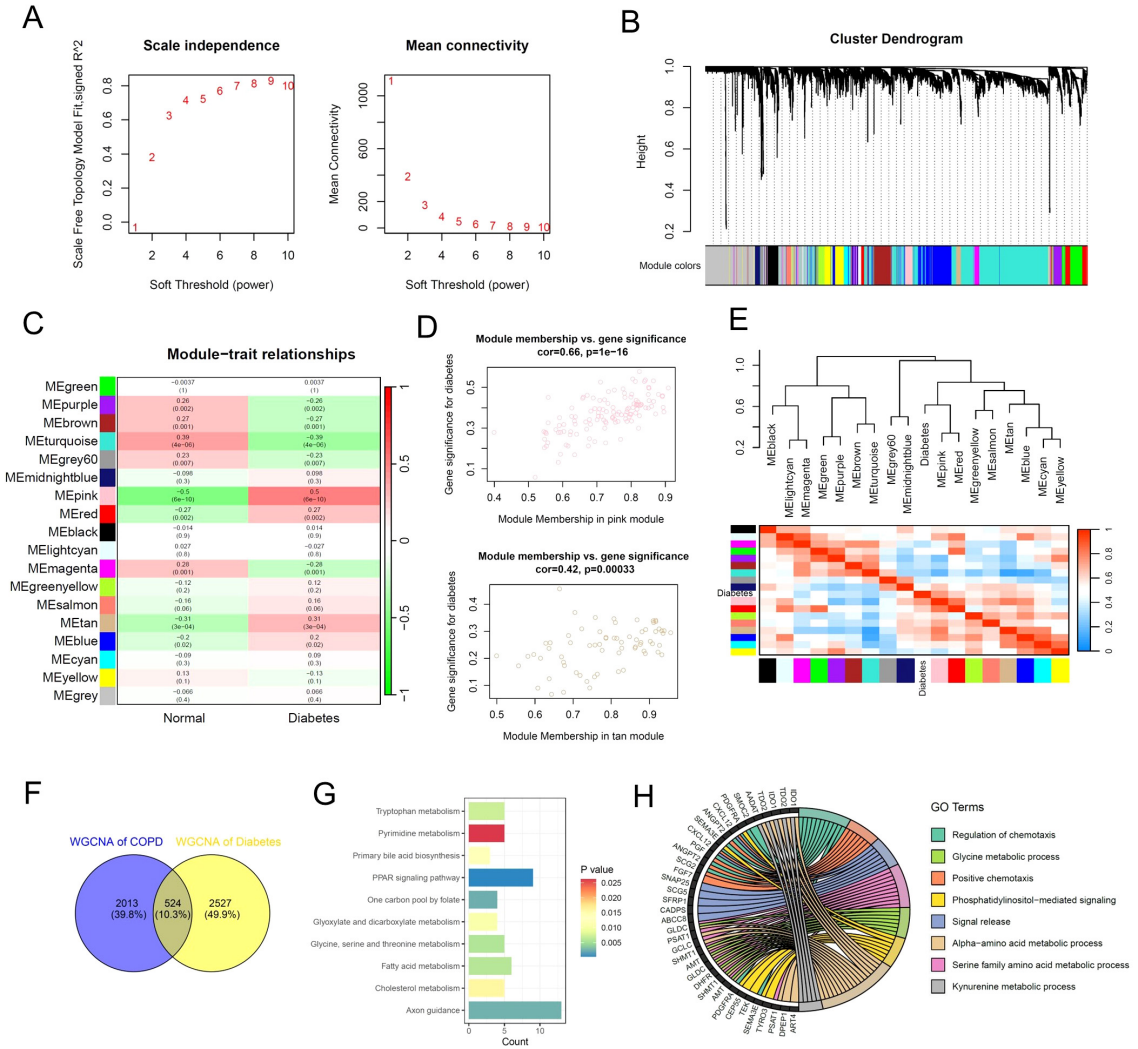
Identification of key gene expression patterns of diabetes using WGCNA algorithm. **(A)** Scale-free topology model identifying the best β value, and β = 7 was chosen as the soft threshold based on the average connectivity and scale independence. **(B)** Cluster dendrogram shows the characteristic genes of different modules. **(C)** Heatmap displays the correlation of different gene modules with diabetes. **(D)** Scatter plot displaying the correlation between representative gene modules and diabetes. **(E)** Hierarchical clustering dendrogram and heatmap illustrating the Pearson correlation between diabetes and various gene modules. **(F)** Venn diagram illustrating key gene modules of WGCNA in the multi-center COPD cohort and diabetes cohort. **(G)** Bar plot displaying the enriched KEGG signaling pathways of the common gene modules of WGCNA. **(H)** Chord diagram displaying the enriched GO signaling pathways of the common gene modules of WGCNA.

The WGCNA results of multi-center diabetes patients showed that the scale-free topology model fit index reached 0.8 when the soft threshold was set to 8 (**Figure 4A**). Similarly, we set the MEDissThres parameter to 0.3 and used the dynamic tree cut algorithm to identify similar gene expression modules. The results showed that 18 modules were identified for subsequent analysis based on the similarity of gene expression (**Figure 4B**). Furthermore, the correlation analysis results between 18 gene expression modules and the diabetes phenotype showed that the pink, red, and tan modules were significantly associated with diabetes, comprising a total of 3051 genes (**Figures 4C, D**). The dendrogram and heatmap results revealed that the pink and red modules were significantly positively correlated with the diabetes phenotype, while the purple module was significantly negatively correlated with the diabetes phenotype (**Figure 4E**). Subsequently, we performed a cross-analysis of the key gene modules related to COPD and diabetes, ultimately identifying 524 key regulatory genes shared between COPD and diabetes (**Figure 4F**). The functional enrichment analysis of the shared 524 key genes revealed the inflammatory and metabolic signaling pathways were involved in COPD and diabetes, consistent with the previous data (**Figures 4G, H**).

### 3.4 Machine learning algorithms identify shared biomarkers for COPD and diabetes

**Figure 5:**
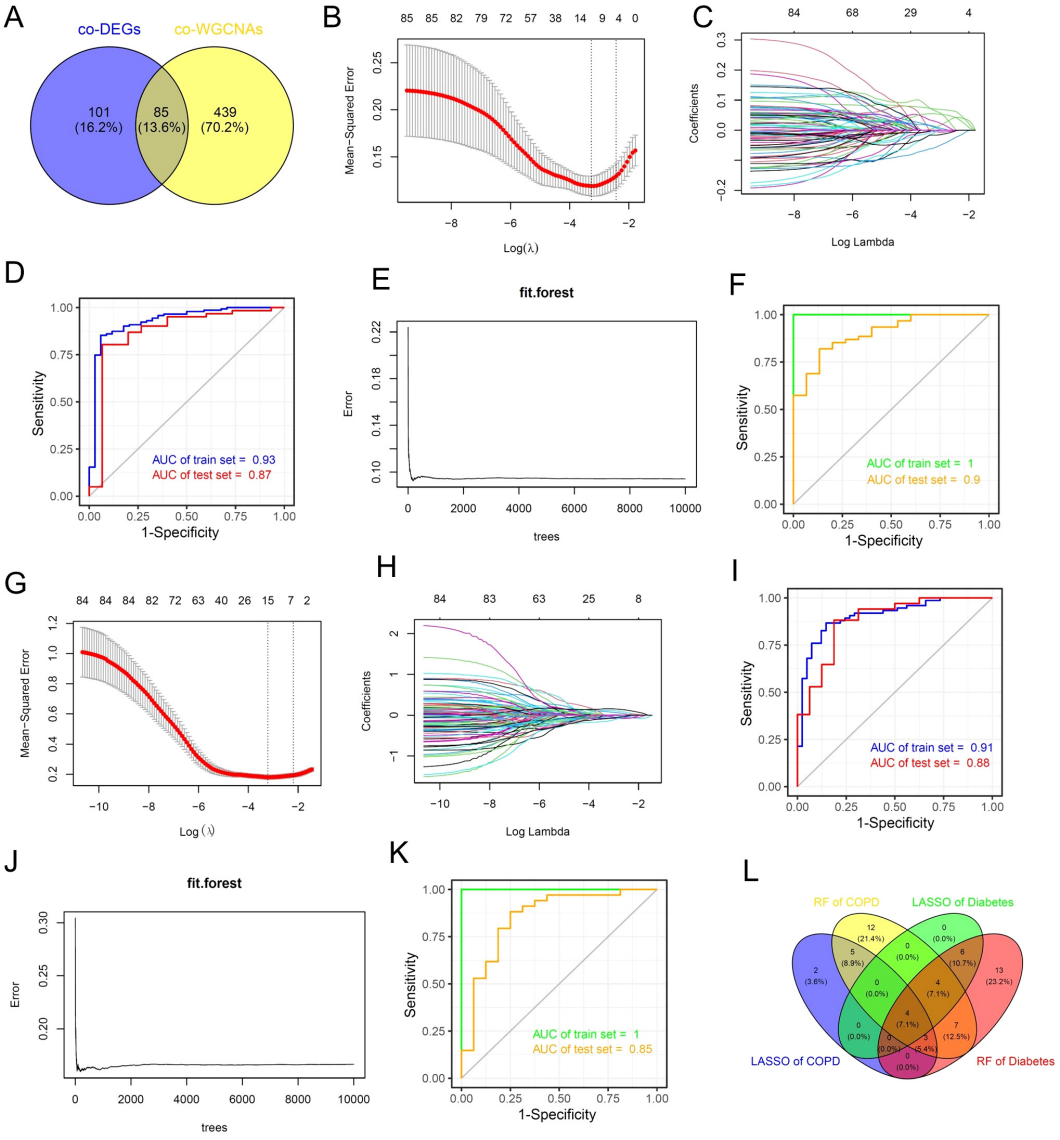
Machine learning algorithms identify shared biomarkers for COPD and diabetes. **(A)** Venn diagram showing the intersection analysis of common DEGs and common WGCNA genes in the COPD and diabetes cohorts. **(B, C)** The lambda values of COPD diagnostic biomarkers identified by the LASSO regression algorithm. **(D)** ROC curve illustrating the predictive efficacy of COPD diagnostic biomarkers identified by LASSO. **(E)** Construction of the random forest model of COPD. **(F)** ROC curve illustrating the predictive efficacy of COPD diagnostic biomarkers identified by random forest. **(G, H)** The lambda values of diabetes diagnostic biomarkers identified by the LASSO regression algorithm. **(I)** ROC curve illustrating the predictive efficacy of diabetes diagnostic biomarkers identified by LASSO. **(J)** Construction of the random forest model of diabetes. **(K)** ROC curve illustrating the predictive efficacy of diabetes diagnostic biomarkers identified by random forest. **(L)** Venn diagram showing the intersection analysis of shared biomarkers identified by LASSO and random forest.

Next, we attempted to integrate various machine learning algorithms to identify potential biomarkers shared between COPD and diabetes. We conducted a cross-analysis of the shared DEGs and shared key genes identified by WGCNA of COPD and diabetes, and ultimately selected 85 potential biomarkers for subsequent machine learning (**Figure 5A**). In brief, we implemented a combination of LASSO and random forest algorithms on multi-center COPD patients and multi-center diabetes patients, respectively. Using the LASSO algorithm, we identified 14 genes as robust biomarkers for COPD patients (**Figures 5B, C**). The results of the ROC analysis showed that the LASSO COX proportional hazards model based on 14 genes was highly robust and accurately predicts the onset of COPD (**Figure 5D**). Through the application of the random forest algorithm, we identified 35 genes as potential biomarkers for COPD patients (**Figure 5E**). The ROC results also indicated that the random forest model constructed based on these 35 genes accurately predicted COPD, whether in the training set or the test set (**Figure 5F**). The same analytical strategy was also used for multi-center diabetes patients. In summary, we identified 15 potential biomarkers for diabetes using the LASSO algorithm and 37 potential biomarkers for diabetes using the random forest algorithm. Both methods accurately predicted diabetes (**Figures 5G–K**). Ultimately, we conducted a cross-analysis of the 4 potential biomarkers gene sets and 4 genes as shared biomarkers for COPD and diabetes, including CADPS, EDNRB, THBS4 and TMEM27 (**Figure 5L**). It is worth mentioning that all of these genes have been reported to be associated with the progression of diabetes (26–29), while EDNRB has been reported to be associated with the onset and progression of COPD (30), demonstrating the reliability of the analytical method.

### 3.5 Shared biomarkers of COPD and diabetes regulate the infiltration of immune cells

**Figure 6:**
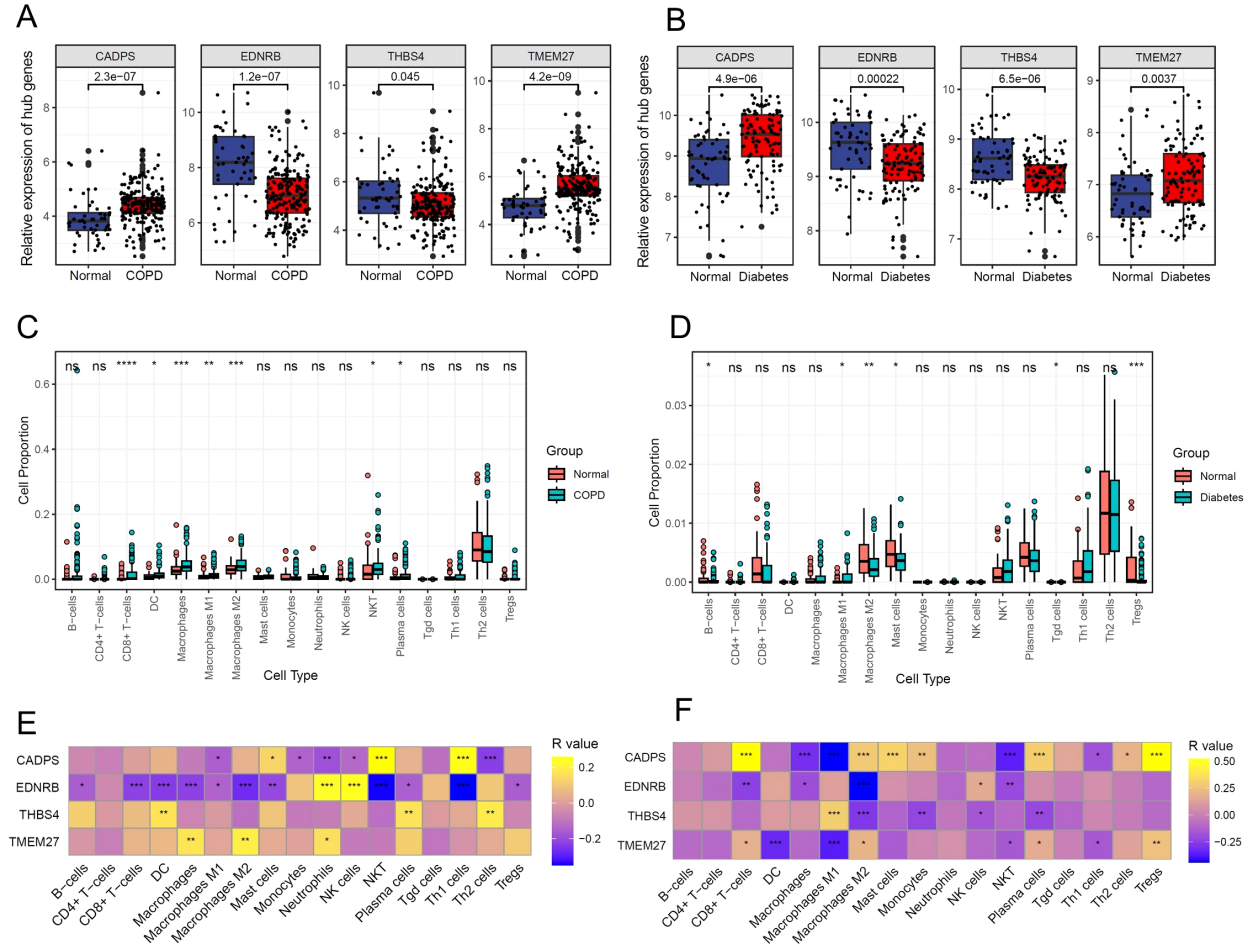
Shared biomarkers of COPD and diabetes regulate the infiltration of immune cells. **(A)** Boxplot displaying the expression of shared biomarkers in COPD and control samples. **(B)** Boxplot displaying the expression of shared biomarkers in diabetes and control samples. **(C)** Boxplot illustrating the infiltration ratio of different immune cells in COPD and control samples. * P<0.05; ** P<0.01; *** P<0.001; **** P<0.0001. **(D)** Boxplot illustrating the infiltration ratio of different immune cells in diabetes and control samples. * P<0.05; ** P<0.01; *** P<0.001; **** P<0.0001. **(E)** Heatmap showing the correlation between shared biomarkers and the infiltration of different immune cells in COPD. **(F)** Heatmap showing the correlation between shared biomarkers and the infiltration of different immune cells in diabetes.

Based on microarray data of multi-center COPD and diabetes patients, the gene expression analysis results showed that CADPS and TMEM27 were significantly upregulated in both COPD and diabetes, while EDNR8 and THBS4 were significantly downregulated (**Figures 6A, B**). Considering the previous analysis revealed the involvement of inflammatory pathways in COPD and diabetes, we further investigated the correlation between shared biomarkers and immune cell infiltration. The immune cell infiltration analysis showed a significant increase in the infiltration of CD8^+^ T cells, dendritic cells (DCs), and macrophages in COPD patients, consistent with previous reports (31–33). Furthermore, B cells, mast cells, and Tgd cells show dysregulation in diabetes, implying a crucial role of immune cells in diabetes (**Figures 6C, D**). The results of the Pearson correlation analysis revealed that the infiltration proportions of multiple immune cells were intricately intertwined with shared biomarkers in COPD and diabetes patients (**Figures 5E, F**). For instance, the expression level of EDNRB was significantly negatively correlated with the infiltration proportion of CD8^+^ T cells, possibly due to the downregulation of EDNRB expression during COPD progression, leading to excessive infiltration of CD8^+^ T cells and triggering an excessive inflammatory response (**Figure 6E**). Similar results were also observed in diabetic patients, where EDNRB was significantly negatively correlated with the infiltration of multiple immune cells, and its abnormal downregulation might induce an excessive inflammatory response (**Figure 6F**).

### 3.6 Shared biomarkers were significantly altered in COPD single-cell atlas

**Figure 7:**
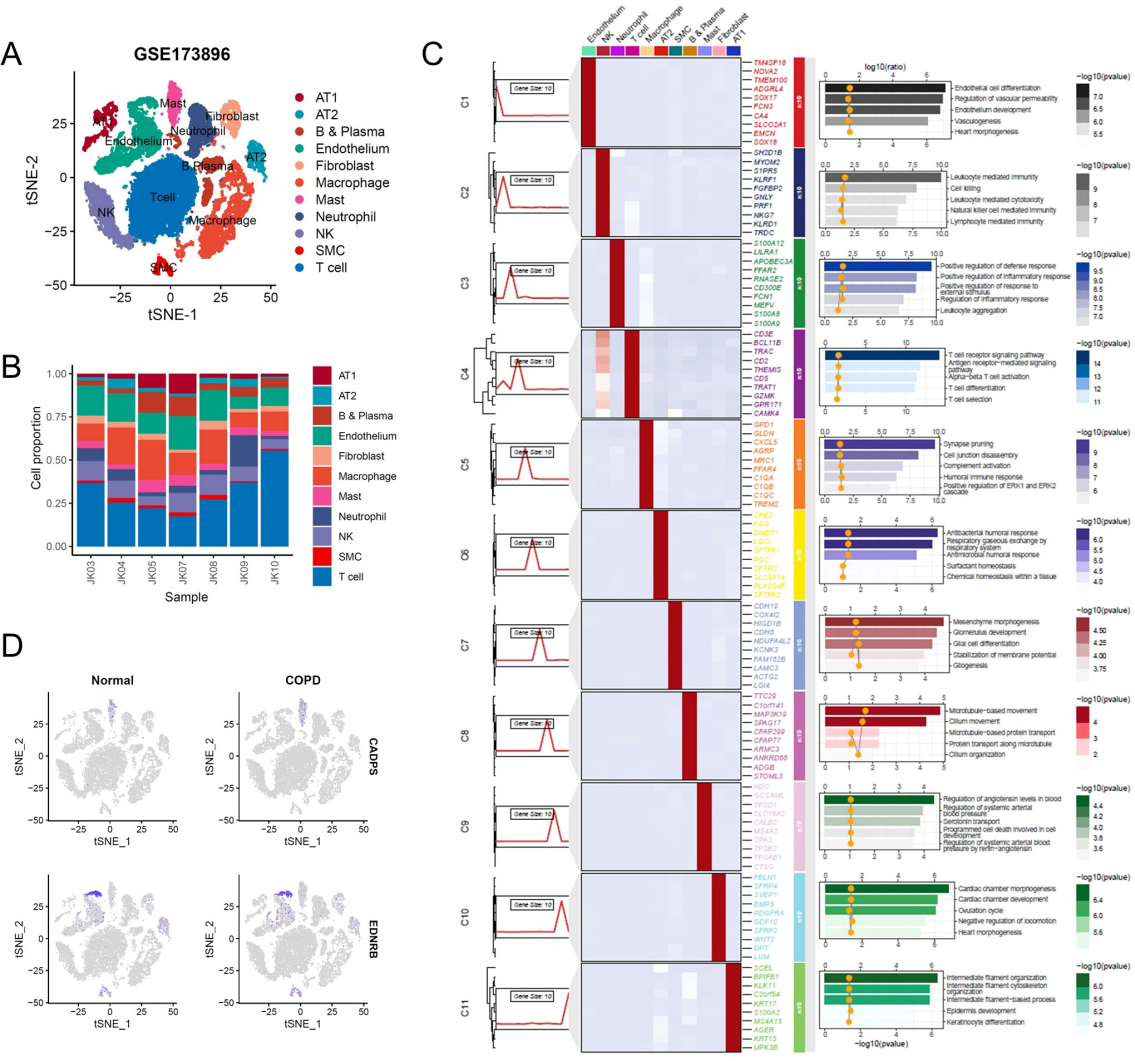
Shared biomarkers were significantly altered in COPD single-cell atlas. **(A)** UMAP plot visualizing the single-cell atlas of COPD patients, identifying distinct cell clusters based on gene expression profiles. Each point represents a single cell, and clusters are color-coded. **(B)** Stacked bar chart showing the relative proportions of various cell types across individual samples, highlighting changes in cellular composition in COPD patients. **(C)** Composite heatmap displaying marker genes and enriched signaling pathways in different cell clusters. **(D)** UMAP plot illustrating the expression levels of shared biomarkers.

To further validate the expression pattern of shared biomarkers in COPD patients, we reanalyzed the single-cell RNA-seq data from COPD patients obtained from GSE173896 (**Figures 7A, B**). Following data processing and cell annotation, we identified 11 distinct cell subclusters, including alveolar type 1 epithelial cells (AT1), which exhibited high expression of genes such as *SCEL* and *BPIFB*, and was enriched in pathways associated with epidermis development; alveolar type 2 epithelial cells (AT1), which exhibited high expression of genes such as *CPB2* and *DMBT1*, and associated with surfactant homeostasis; alveolar type 2 epithelial cells (AT1), which exhibited high expression of genes such as *CPB2* and *DMBT1*, and associated with surfactant homeostasis (**Figure 7C**). Next, we investigated the expression of shared biomarkers in single cells. Consistent with previous analysis, CADPS was significantly highly expressed in macrophages, while EDNRB was significantly highly expressed in endothelial cells. Furthermore, CADPS was significantly upregulated in COPD, while EDNRB was significantly downregulated in COPD (**Figure 7D**). The above results indicated that shared biomarkers demonstrated dysregulation across several COPD patient cohorts and multi-omics, making them robust biomarkers for COPD.

### 3.7 Shared biomarkers were significantly altered in diabetes single-cell atlas

**Figure 8:**
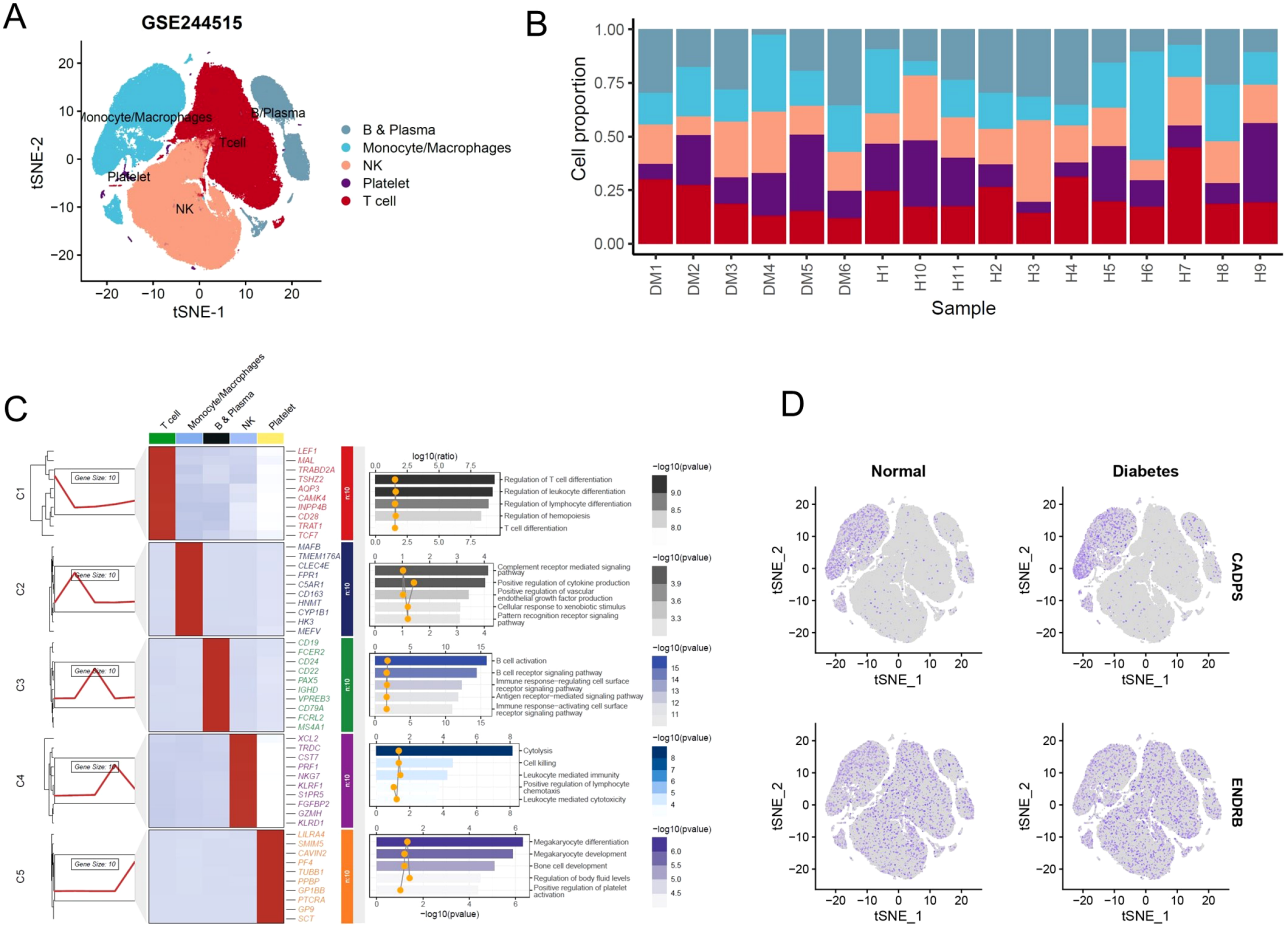
Shared biomarkers were significantly altered in diabetes single-cell atlas. **(A)** UMAP plot visualizing the single-cell atlas of diabetes patients, identifying distinct cell clusters based on gene expression profiles. Each cluster is color-coded to represent a specific cell type. **(B)** Stacked bar chart displaying the proportions of various cell types across individual diabetes samples, showing notable shifts in immune and metabolic cell populations. **(C)** Composite heatmap displaying marker genes and enriched signaling pathways in different cell clusters. **(D)** UMAP plot illustrating the expression levels of shared biomarkers.

Similarly, we re-integrated and analyzed single-cell sequencing data from peripheral blood of diabetic patients to observe the predictive value of shared biomarkers in diabetes (**Figures 8A, B**). We ultimately identified 5 distinct cell clusters, including B and plasma cells, with high expression of *CD19*, *CD79A*, and involvement in receptor-related immune cell activation; monocytes and macrophages, with high expression of *MAFB*, *CD163*, and involvement in the signaling pathways of complement activation and immune factor release; natural killer (NK) cells, with high expression of *NKG7*, *KLRF1*, and involvement in cell killing; platelets, with high expression of *LILRA4*, *TUBB1*, and involvement in megakaryocyte differentiation; T cells, with high expression of LEFA, TRAT1, and involvement in T cell-mediated cell activation (**Figure 8C**). As expected, CADPS was observed to be highly expressed in macrophages and significantly increased in diabetic patients, making it a robust biomarker for diabetes patients (**Figure 8D**). Conversely, the expression level of ENDRB significantly decreased in diabetic patients, implying that the functional inactivation of ENDRB was crucial for the development of diabetes (**Figure 8D**). In summary, shared biomarkers exhibited good predictive value in multi-center microarray data of diabetic patients, and demonstrated considerable consistency in single-cell RNA-seq, thus serving as robust biomarkers for diabetic patients.

### 3.8 Validation of shared biomarkers in human tissues and mouse models

**Figure 9:**
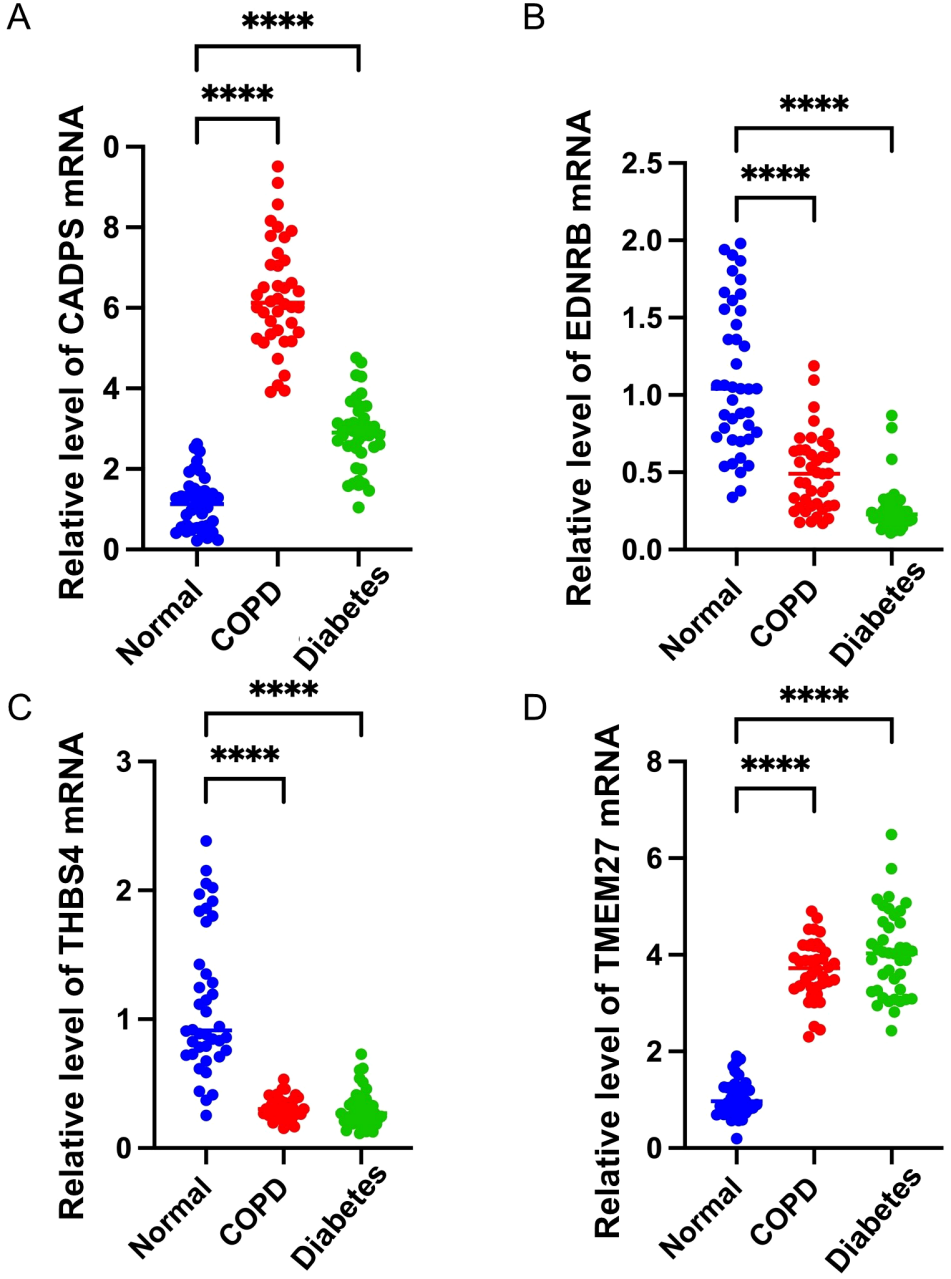
Validation of shared biomarkers in clinical samples via RT-qPCR assays. **(A-D)** The mRNA expression levels of shared biomarkers (CADPS, EDNRB, THBS4, TMEM27) were measured in serum samples from healthy individuals, COPD patients, and diabetes patients. The boxplots illustrate significant upregulation of CADPS and TMEM27 and significant downregulation of EDNRB and THBS4 in disease groups compared to healthy controls. These results confirm the robustness of the bioinformatic analysis. **** P<0.0001.

**Figure 10:**
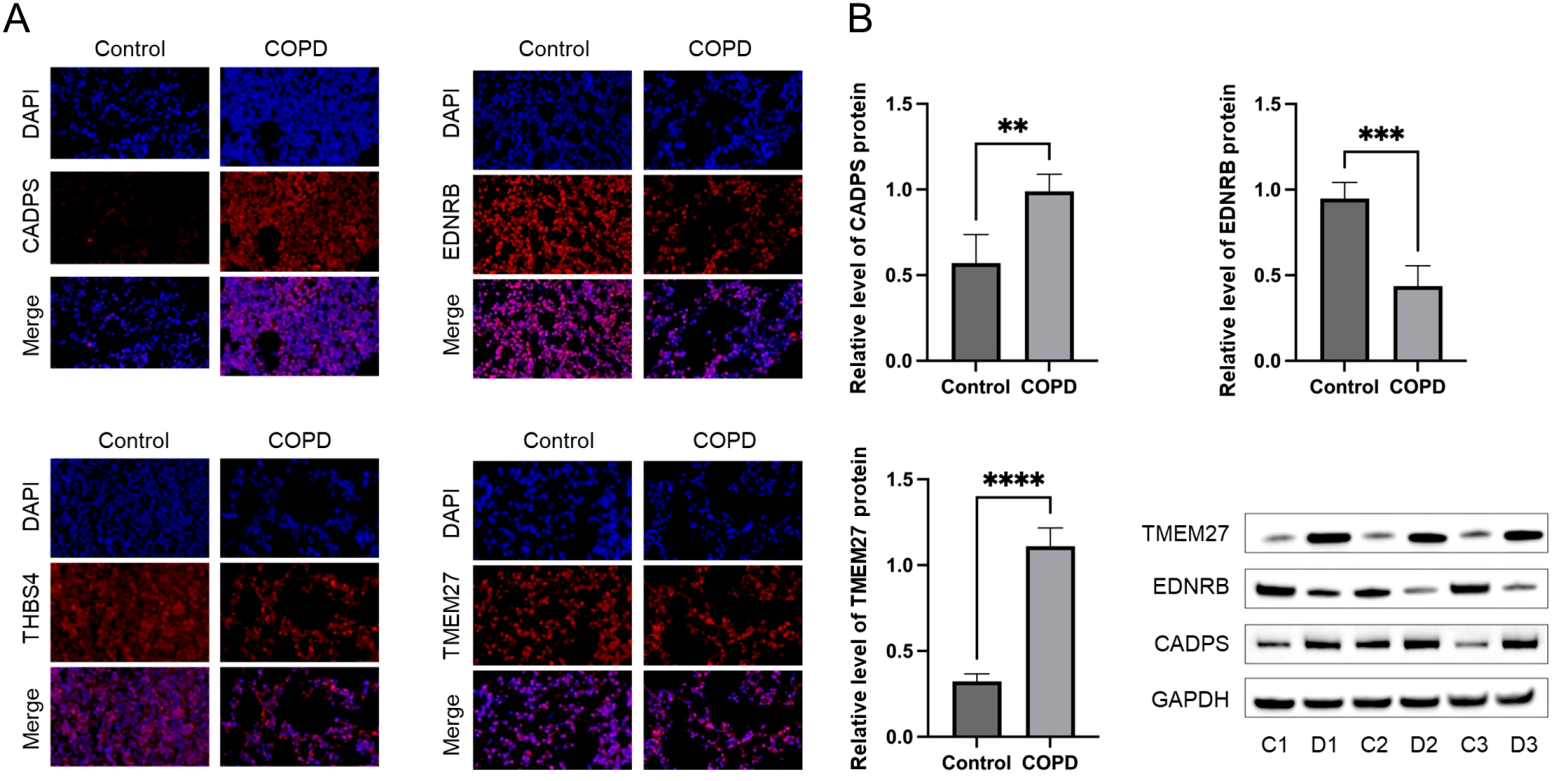
Validation of shared biomarkers in COPD models via IF and WB assays. **(A)** Representative IF images showing the expression of shared biomarkers (CADPS, EDNRB, TMEM27) in lung tissues from control and COPD mice. CADPS and TMEM27 were significantly upregulated, while EDNRB was downregulated in COPD tissues. **(B)** WB analysis quantifying the protein levels of shared biomarkers in lung tissues, confirming the trends observed in IF staining and validating their role in COPD progression. ** P<0.01; *** P<0.001; **** P<0.0001.

**Figure 11:**
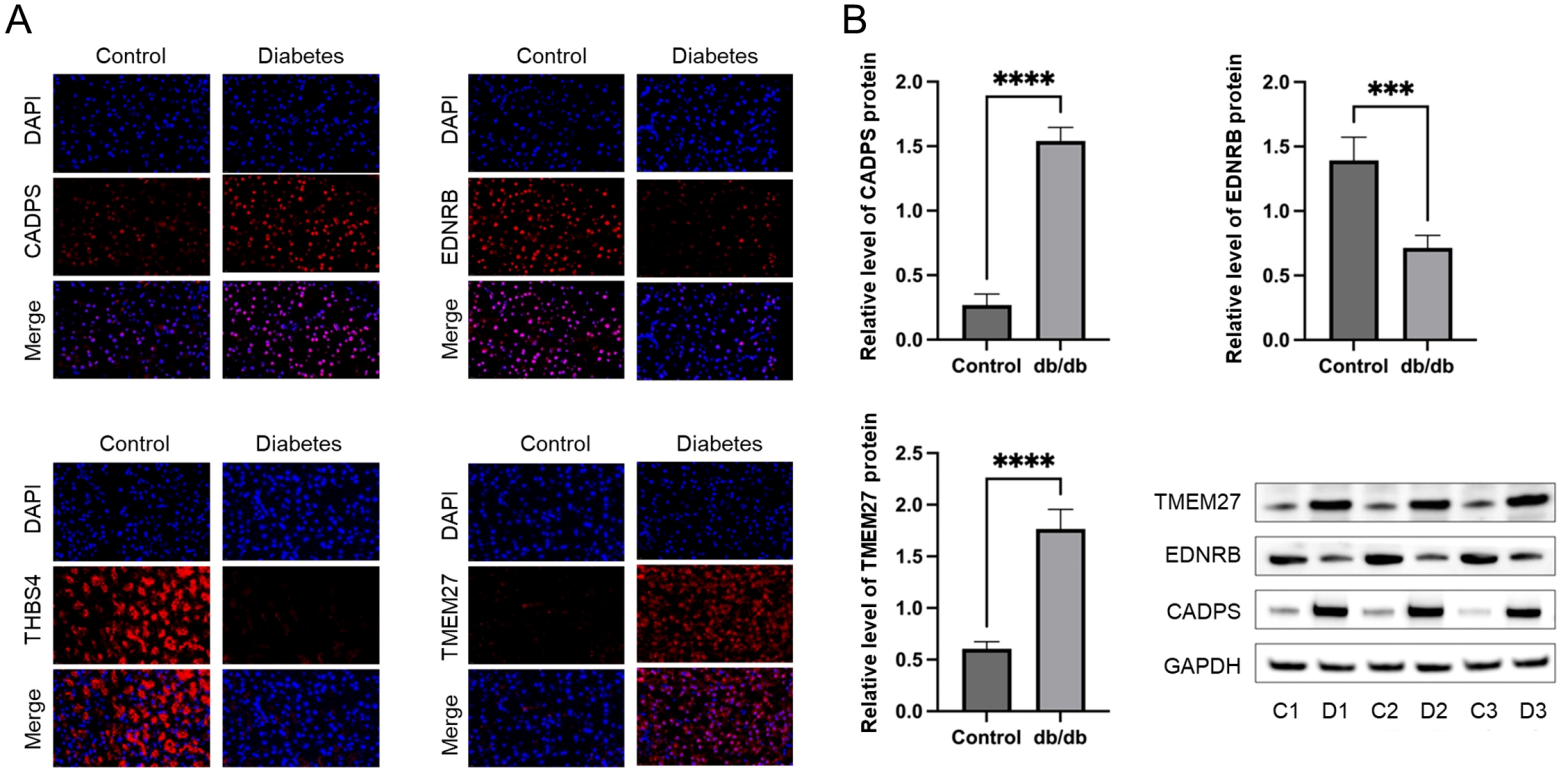
Validation of shared biomarkers in diabetes models via IF and WB assays. **(A)** Representative IF images showing the expression of shared biomarkers in pancreatic tissues from control and diabetes mice. CADPS and TMEM27 were significantly upregulated, while EDNRB was downregulated in diabetes tissues. **(B)** WB analysis quantifying the protein levels of shared biomarkers in pancreatic tissues, aligning with the IF results and validating their involvement in diabetes pathophysiology. *** P<0.001; **** P<0.0001.

To validate the shared biomarkers identified by machine learning, we gathered serum samples from 40 healthy individuals, 40 COPD patients, and 40 diabetic patients for *in vitro* testing. The results of RT-qPCR demonstrated that, compared to healthy individuals, the serum of COPD and diabetic patients exhibited significant increases in CADPS and TMEM27, and significant decreases in EDNRB and THBS4, consistent with the results of bioinformatics analysis (**Figures 9A–D**). Furthermore, based on previous reports, we established mouse models of COPD and diabetes, and extracted lung and pancreatic tissues to detect the expression of shared biomarkers. The results of IF and WB assays indicated that, compared to the control group, the lung tissue of the COPD mice displayed a significant increase in CADPS and TMEM27, and a significant decrease in EDNRB and THBS4 (**Figures 10A, B**). The assessment of pancreatic tissue in the diabetic mouse model also revealed similar results (**Figures 11A, B**). The above results demonstrated that the shared biomarkers exhibit sensitivity and robustness in both clinical samples and animal models, allowing for precise prediction of the progression of COPD and diabetes.

## 4 Discussion

Diabetes is a chronic metabolic disease and steadily increasing worldwide, characterized by abnormally high blood glucose levels, mainly resulting from inadequate insulin secretion or insulin resistance. The etiology of diabetes is complex, involving multiple factors including genetics, environment, and lifestyle. Genetic factors play a significant role in the onset of diabetes, where variations in certain genes can increase the risk of diabetes. Environmental factors such as overnutrition, insufficient physical activity, and obesity are also closely linked to the onset of diabetes. Based on different etiologies and clinical presentations, diabetes is mainly classified into type 1 diabetes, type 2 diabetes, gestational diabetes, and other specific types of diabetes. The complications of diabetes involve multiple systems, including the cardiovascular system, nervous system, kidneys, and eyes. Among these, microvascular changes are one of the most common complications of diabetes, such as diabetic retinopathy and diabetic nephropathy.

Some studies have revealed a significant increase in the risk of COPD in patients with diabetes (13). This association may involve multiple factors such as inflammatory mediators, weight control, and metabolic abnormalities. Additionally, some studies have indicated that the incidence of diabetes is also higher among COPD patients, suggesting a potential common pathophysiological mechanism between COPD and diabetes. For instance, a prospective cohort study observed that compared to the control group, the risk of developing COPD increased by 18% and 35% for prediabetic individuals and diabetic patients, respectively, suggesting a potential shared pathogenic mechanism between diabetes and COPD (12). However, it is regrettable that the shared pathogenic mechanisms and biomarkers between diabetes and COPD have not yet been identified to accurately predict the onset risk in patients. In this study, we integrated microarray data from multiple cohorts of COPD and diabetes patients, and comprehensively utilized various machine learning algorithms to identify shared biomarkers in COPD and diabetes patients, including CADPS, EDNRB, THBS4 and TMEM27.

CADPS is a Ca^2+^ dependent secretory protein, which plays an important role in vesicle fusion on the presynaptic membrane of neurons (34). In recent years, CADPS protein has been reported to interact with the SNARE protein family, regulating neurotransmitter release, particularly in the regulation of insulin secretion and neurotransmitter release (35). For instance, it is reported that the decreased insulin content and granule is related to lysosome quantity and lysosomal enzyme activity in β cells of CADPS conditional knockout mice. CADPS has been identified as crucial regulators of insulin granule initiation, exocytosis, and stability (36). In this study, we observed that the expression level of CADPS is abnormally increased in diabetic patients, leading to the disruption of metabolic balance in pancreatic β cells and participating in the pathogenesis of diabetes. Furthermore, analogous alterations have been verified in COPD patients, indicating a potential correlation between diabetes and COPD. Despite the unreported role of CADPS in COPD progression, our findings indicate its potential correlation with alterations in immune microenvironment of COPD. Correlation analysis results demonstrate a significant positive correlation between CADPS and mast cell infiltration in COPD tissues, which has been reported as a significant cause of COPD onset (37).

EDNRB is a G protein-coupled receptor which binds to endothelin, exerting significant effects on vasoconstriction, cell proliferation, and inflammatory responses (27, 38). Recently, the role of EDNRB in diabetes and COPD has become a research focus (39, 40). For instance, it has reported that insulin upregulates the expression of endothelial cell EDNRB through the PI3K/Akt pathway, reducing the acceleration of diabetic atherosclerosis (27). In addition, celastrol has been reported to regulate the expression of EDNRB and alleviate COPD by inhibiting inflammation development (38). In this study, we identified EDNRB as a shared biomarker for diabetes and COPD, and accurately predicted the diagnosis of diabetes and COPD. Our results mutually corroborate existing reports, suggesting that inhibiting EDNRB may aid in alleviating diabetic complications and improving respiratory function.

THBS4 is a multifunctional extracellular matrix protein of the thrombospondin family, participating in tissue repair and the inflammation cascade of various diseases (41, 42). Although the molecular mechanisms of THBS4 in diabetes and COPD have not been fully elucidated, it has been identified as a potential risk factor for diabetes in existing reports, which may be related to its regulation of the immune microenvironment (28). For example, it has reported that proliferation, migration, inflammation, and differentiation-related signaling pathways in keratinocytes are significantly activated after being stimulated by THBS4 (43). Furthermore, complement C3a treatment stimulated overall white matter reorganization, upregulating the expression of THBS4 in the peri-infarct cortex of ischemic stroke, thereby promoting inflammatory responses and tissue repair (44). Our results revealed that THBS4 reshapes the immune microenvironment by interfering with the immune infiltration of DC cells and macrophages, and ultimately regulates the progression of diabetes and COPD. While the molecular mechanisms of THBS4 in diabetes and COPD still require further clarification, intervening in THBS1 may be an effective therapeutic strategy.

TMEM27 is a protein mainly expressed in pancreatic islet cells, promoting the regeneration of β cells by influencing cell cycle proteins and growth factor signals (29, 45). Additionally, TMEM27 has been reported to be involved in the transport, absorption, and metabolism of amino acids, playing a significant role in maintaining normal kidney function (46). Our results revealed a significant increase in the expression levels of TMEM27 in patients with COPD and diabetes. Furthermore, we found that TMEM27 is associated with the infiltration of various immune cells, such as macrophages and T cells, providing a theoretical basis for exploring the function of TMEM27.

Recent evidence underscores the importance of systemic inflammation and metabolic dysregulation as shared mechanisms in COPD and diabetes. For example, elevated levels of pro-inflammatory cytokines (IL-1β, TNF-α) contribute to insulin resistance and airway remodeling, creating a bidirectional feedback loop that exacerbates disease progression (13). Furthermore, NRF2 dysfunction, a common feature in both diseases, highlights the role of oxidative stress and mitochondrial dysfunction in linking these conditions (14). Biomarkers such as EDNRB and THBS4 have been validated in other multimorbidity studies, demonstrating their roles in vascular inflammation and immune regulation (4). These findings are consistent with our identification of these genes as shared regulatory nodes in both diseases. Additionally, the integration of multi-omics data, as demonstrated in recent studies (17, 18), has further supported the utility of approaches like ours in uncovering shared biomarkers.

To further explore biological implications, we have validated the expression patterns of these biomarkers in COPD and diabetes animal models. The consistent upregulation or downregulation observed in lung and pancreatic tissues supports their functional relevance in these diseases. While prior studies have explored the role of EDNRB and TMEM27 in either diabetes or COPD, our study is among the first to leverage multi-omics data to identify these biomarkers as shared regulatory nodes between the two diseases. This approach highlights their dual relevance and suggests potential common pathways driving the co-progression of diabetes and COPD. Beyond confirming the involvement of EDNRB and TMEM27 in diabetes or COPD, our study reveals their interactions with immune cell infiltration and metabolic pathways in both diseases.

While this study provides compelling evidence for the role of CADPS, EDNRB, THBS4, and TMEM27 as shared biomarkers between COPD and diabetes, it is important to acknowledge that the specific molecular mechanisms underlying their involvement in disease progression remain incompletely understood. The identification of these biomarkers through statistical and machine learning approaches offers valuable insights, but their precise molecular behaviors and interactions within the pathological contexts of COPD and diabetes require further elucidation. Future studies should focus on experimental validation of these biomarkers using molecular and cellular techniques, such as gene knockdown or overexpression models, to determine their causal roles in disease pathways. For instance, exploring how CADPS modulates β-cell function in diabetes or immune cell activation in COPD through *in vitro* and *in vivo* experiments could clarify its functional relevance. Similarly, investigating the downstream signaling pathways regulated by EDNRB and THBS4 in inflammatory and vascular contexts could provide a mechanistic basis for their association with disease. Furthermore, while this study integrates multi-omics data and provides robust statistical correlations, the translational potential of these biomarkers in clinical practice would benefit from large-scale cohort studies and interventional trials to validate their diagnostic and therapeutic applicability. Understanding the interplay of these biomarkers at the molecular level will not only solidify their biological significance but also pave the way for novel therapeutic strategies targeting shared pathways in COPD and diabetes.

In addition, the datasets used in this study predominantly comprised participants of European ancestry. While this homogeneity reduces inter-population variability, it may limit the generalizability of our findings to other populations. While our study highlights robust associations between the identified biomarkers and disease progression, further validation in larger, independent cohorts is necessary to strengthen these findings. Such studies should also explore population-specific variations in biomarker expression to ensure the global applicability of these results.

In summary, our study integrated multiple COPD and diabetes cohorts and identified shared biomarkers through various machine learning algorithms. Furthermore, we validated the robustness of these shared biomarkers in single-cell sequencing data, clinical samples from patients, and animal models. Our study revealed the potential connection between diabetes and COPD, providing a theoretical basis for exploring the common regulatory genes.

## Data Availability

The original contributions presented in the study are included in the article/**Supplementary Material**. Further inquiries can be directed to the corresponding author.
