# Supplementary figures and images for: Comprehensive bioinformatics analysis identifies metabolic and immune-related diagnostic biomarkers shared between diabetes and COPD using multi-omics and machine learning
Source: Front Endocrinol (Lausanne). 2025 Jan 8;15:1475958. doi: 10.3389/fendo.2024.1475958 (PMC11750655; doi:10.3389/fendo.2024.1475958)

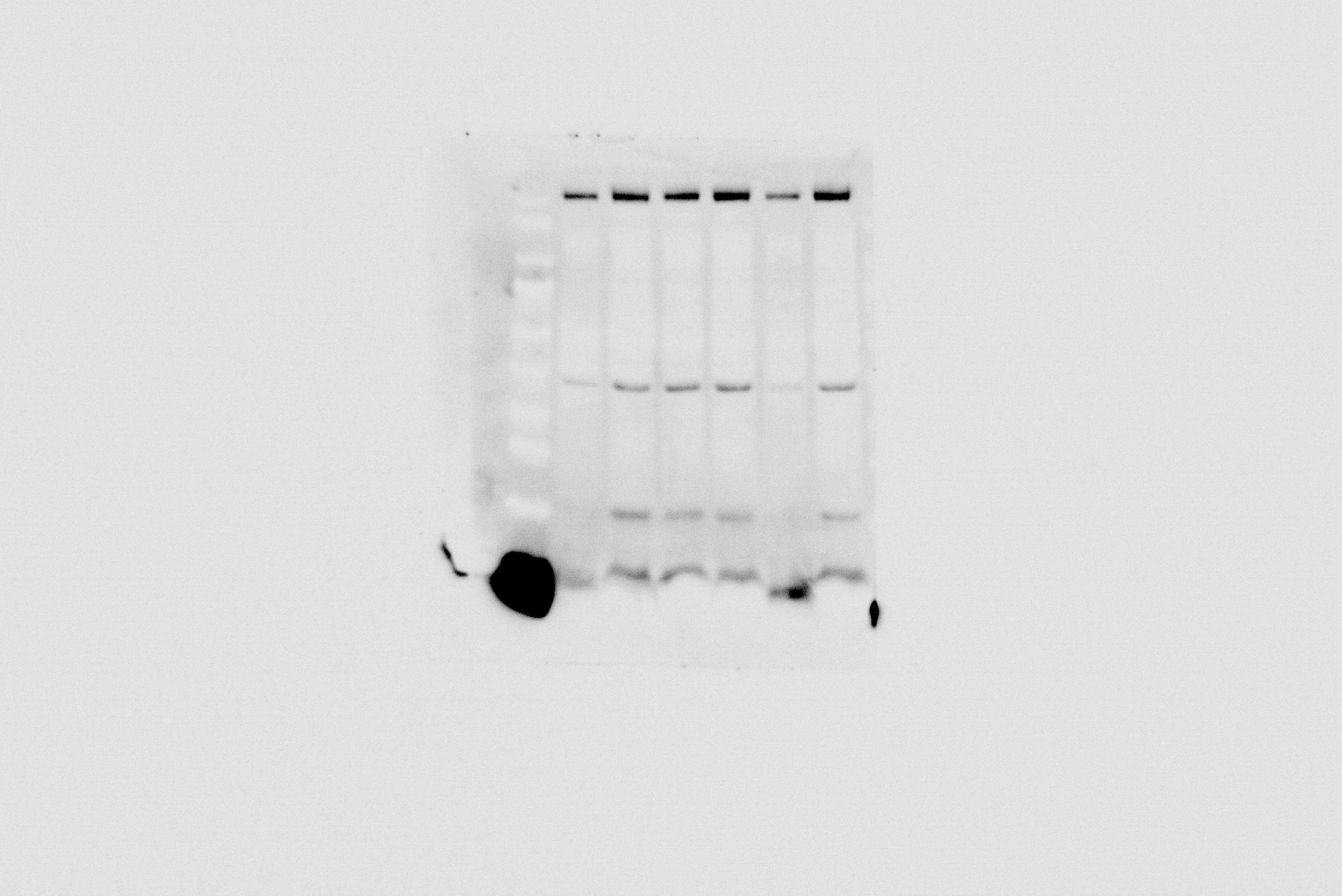

Supplement: Supplementary file 1 [file DataSheet1.zip › original image files for WB/Lung/cadps-lung/CAPS1.jpg]

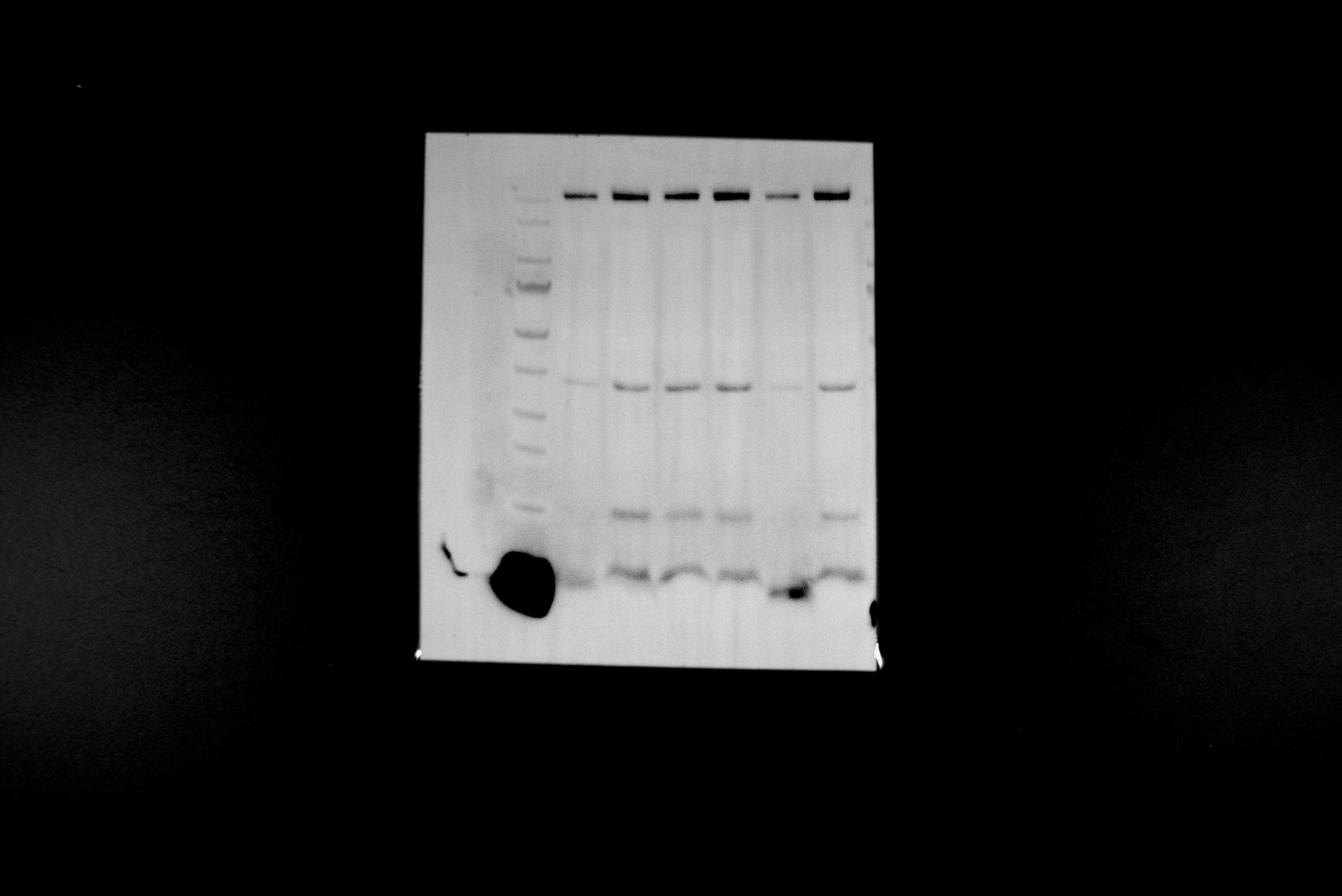

Supplement: Supplementary file 1 [file DataSheet1.zip › original image files for WB/Lung/cadps-lung/CAPS2.jpg]

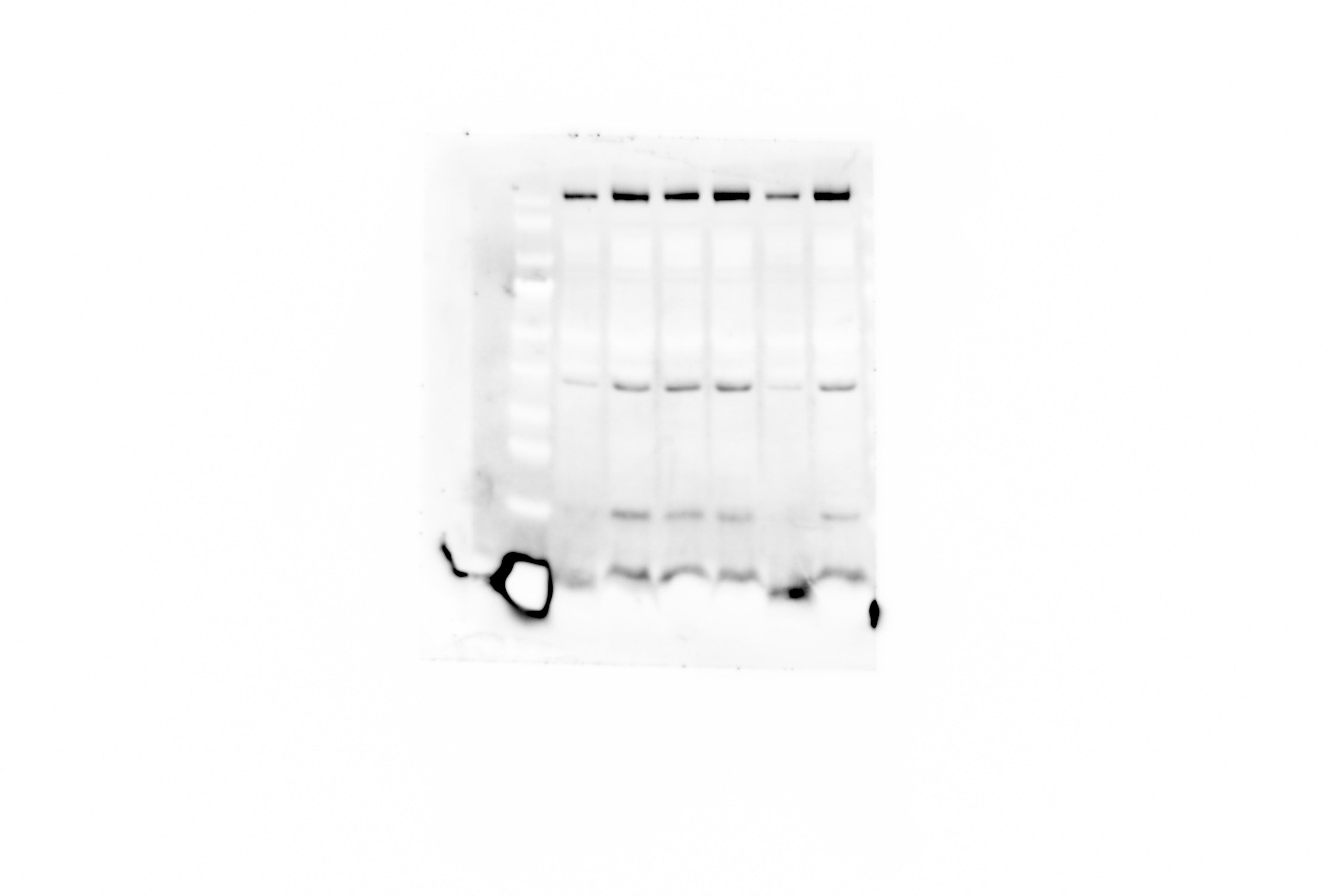

Supplement: Supplementary file 1 [file DataSheet1.zip › original image files for WB/Lung/cadps-lung/CAPS3.jpg]

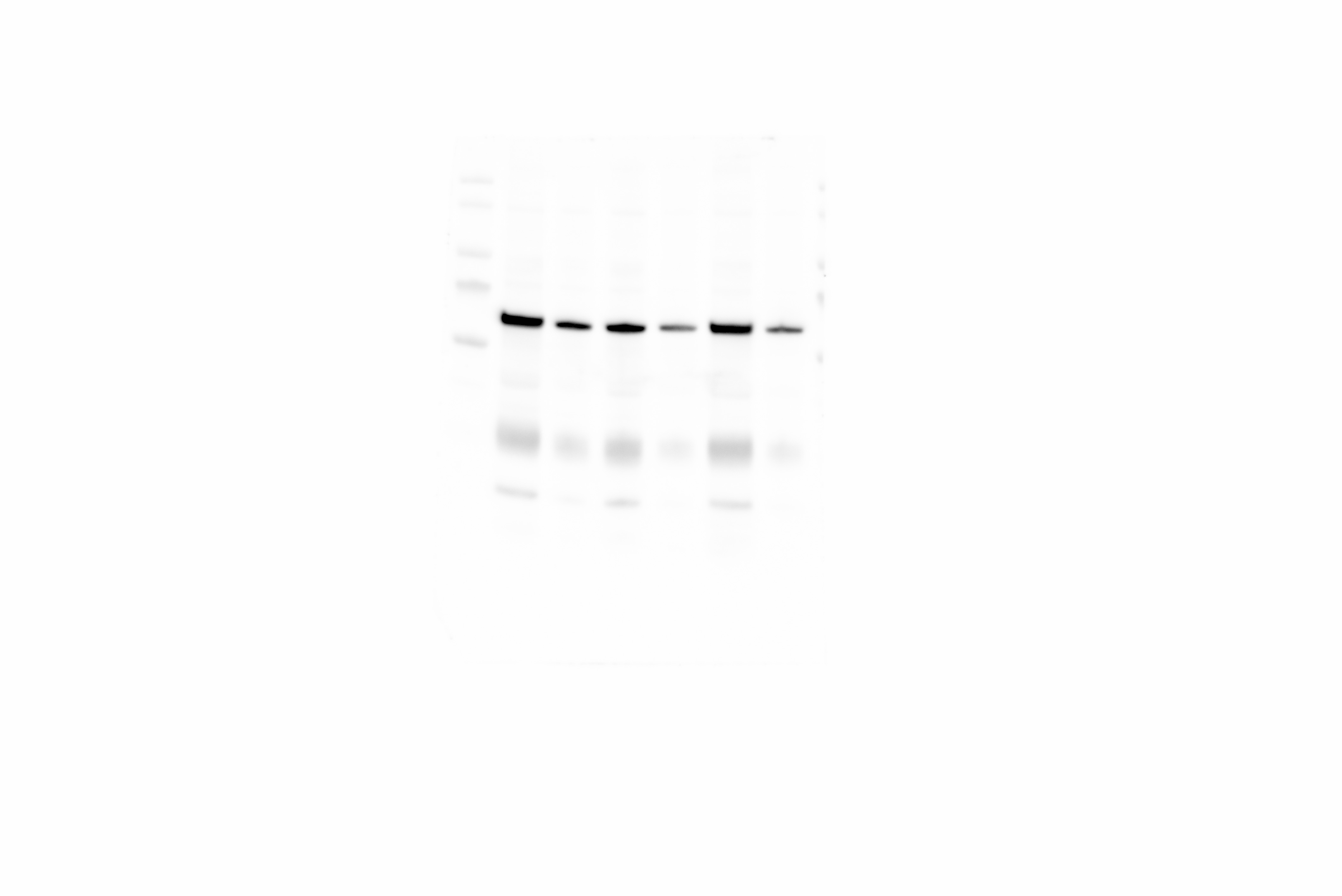

Supplement: Supplementary file 1 [file DataSheet1.zip › original image files for WB/Lung/ednrb-lung/EDNRB.jpg]

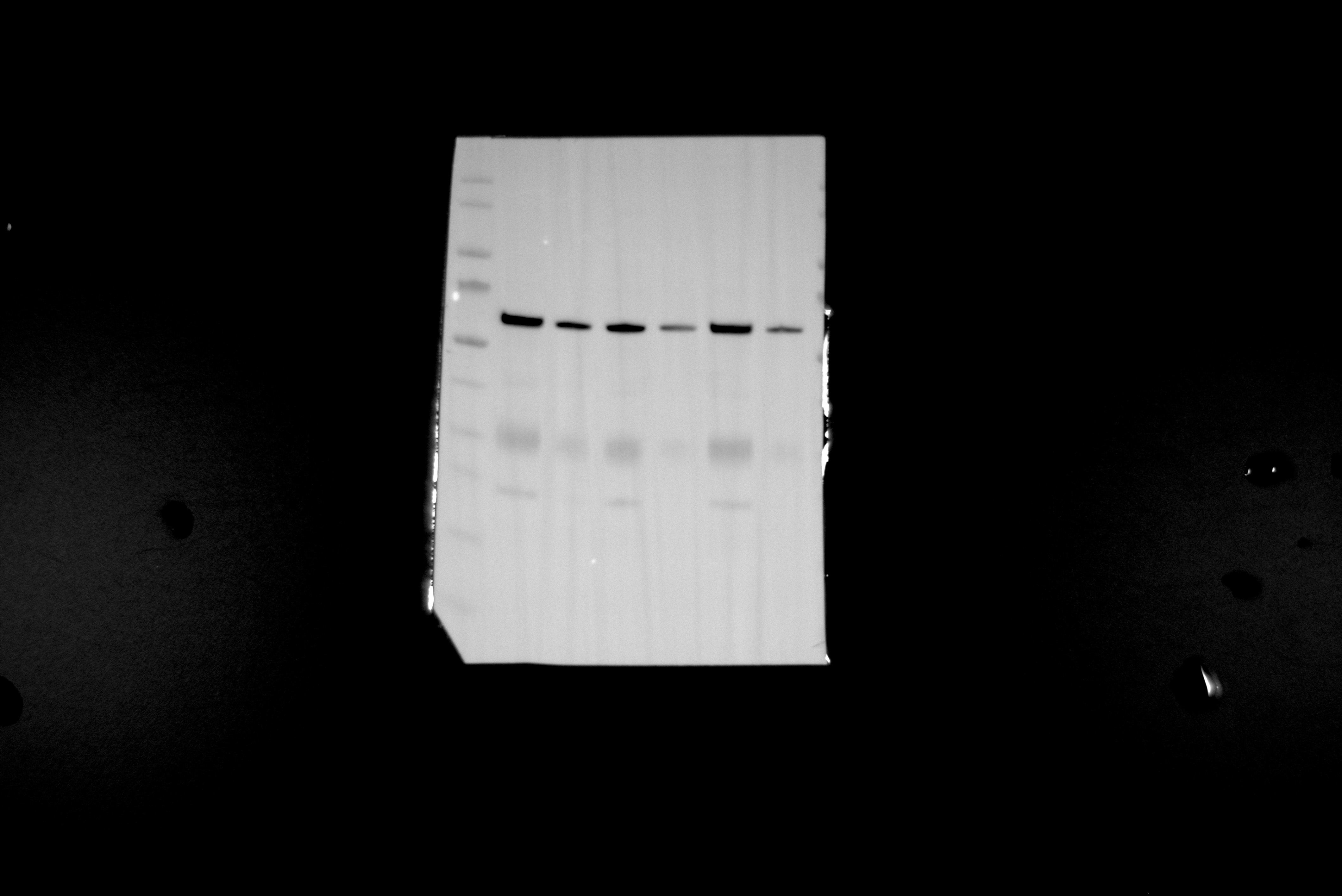

Supplement: Supplementary file 1 [file DataSheet1.zip › original image files for WB/Lung/ednrb-lung/EDNRB1.jpg]

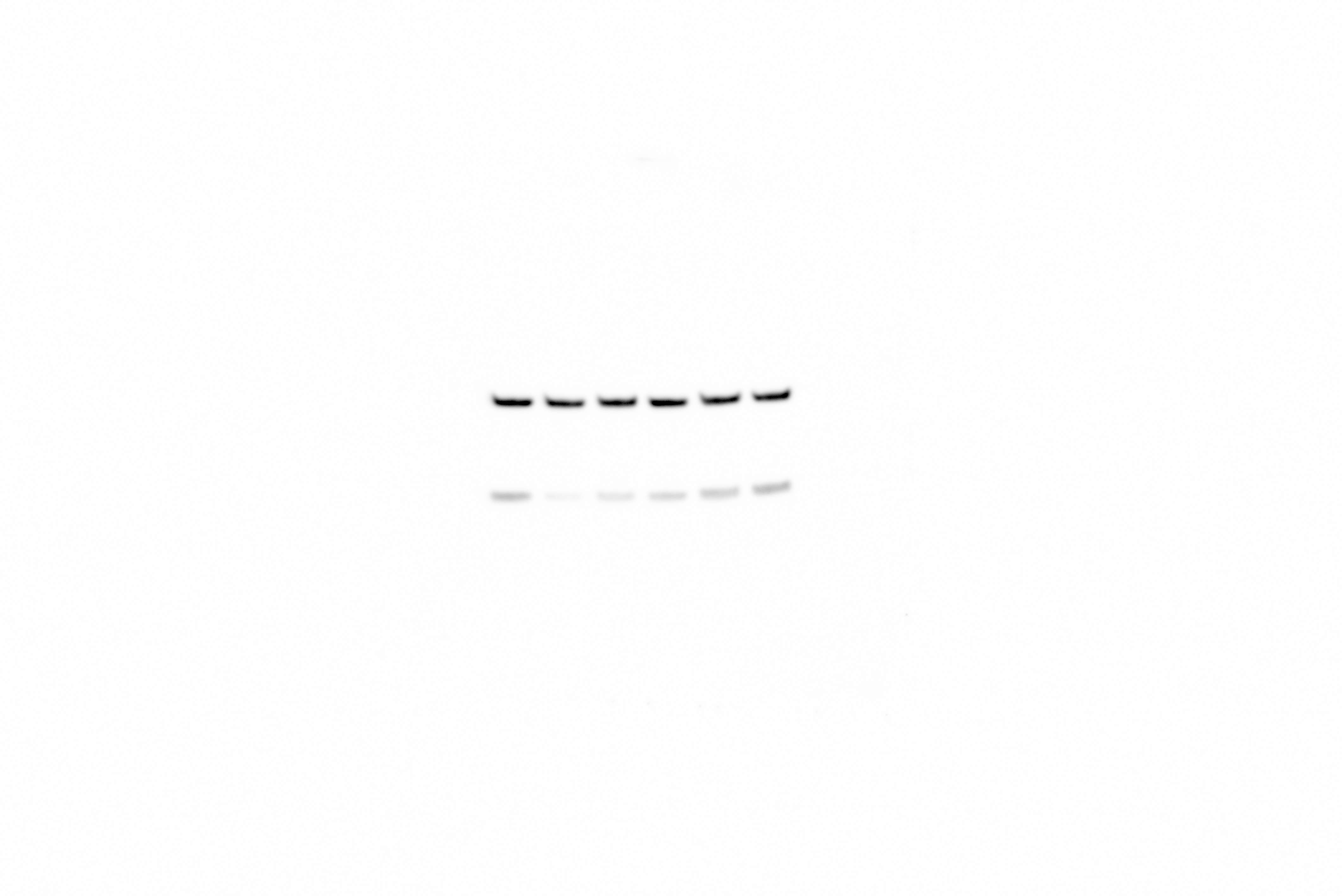

Supplement: Supplementary file 1 [file DataSheet1.zip › original image files for WB/Lung/gapdh-lung/GAPDH.jpg]

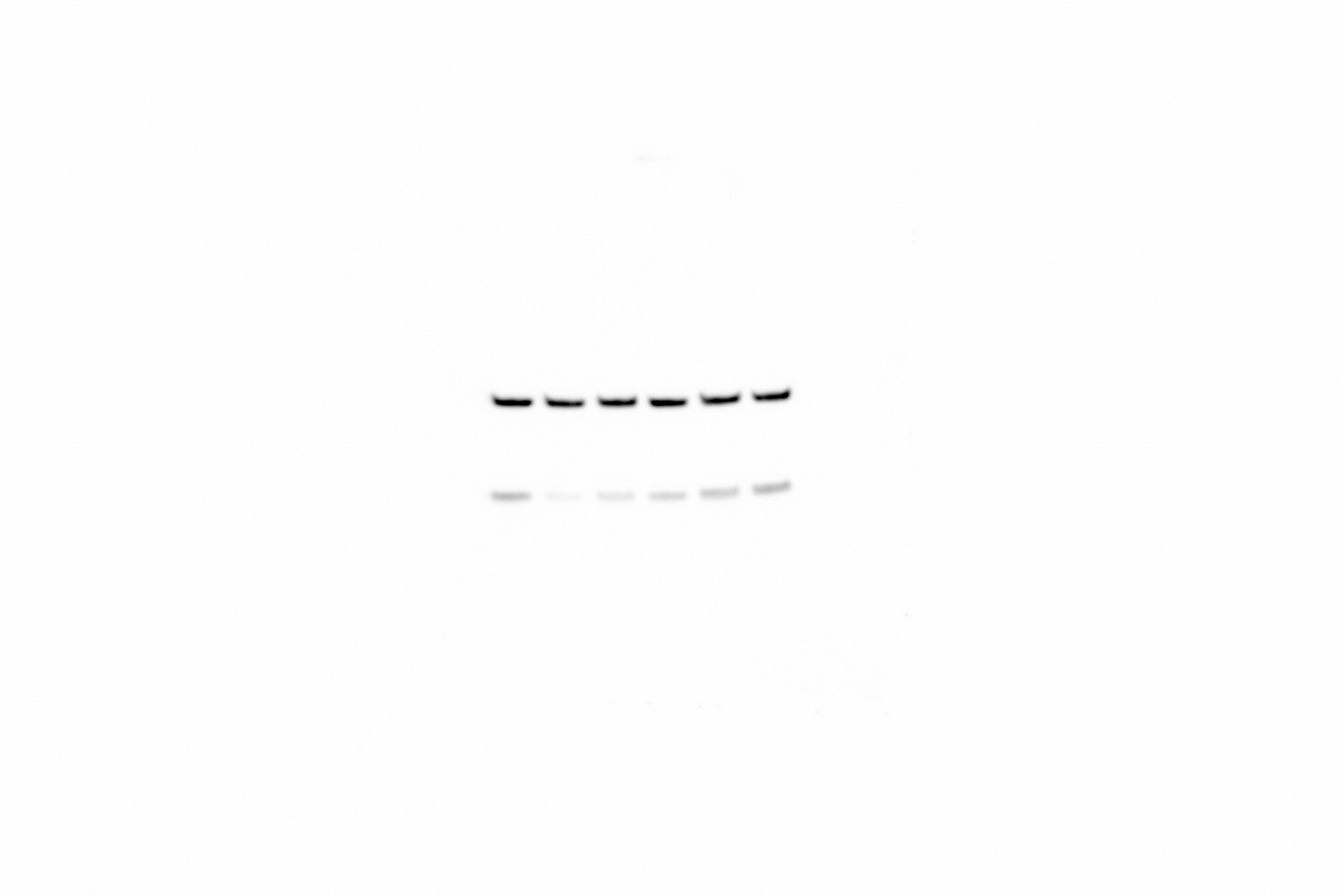

Supplement: Supplementary file 1 [file DataSheet1.zip › original image files for WB/Lung/gapdh-lung/GAPDH1.jpg]

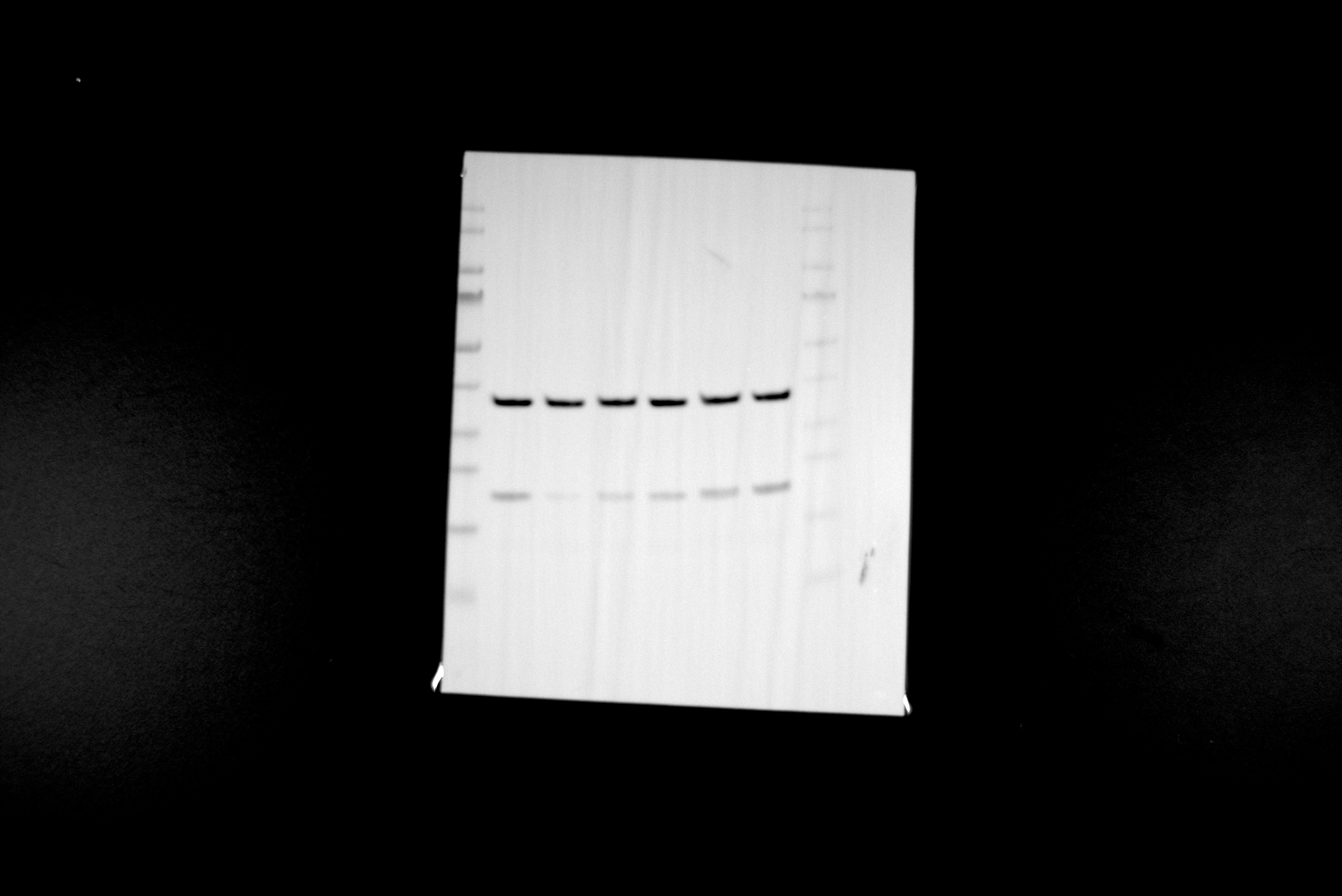

Supplement: Supplementary file 1 [file DataSheet1.zip › original image files for WB/Lung/gapdh-lung/GAPDH12.jpg]

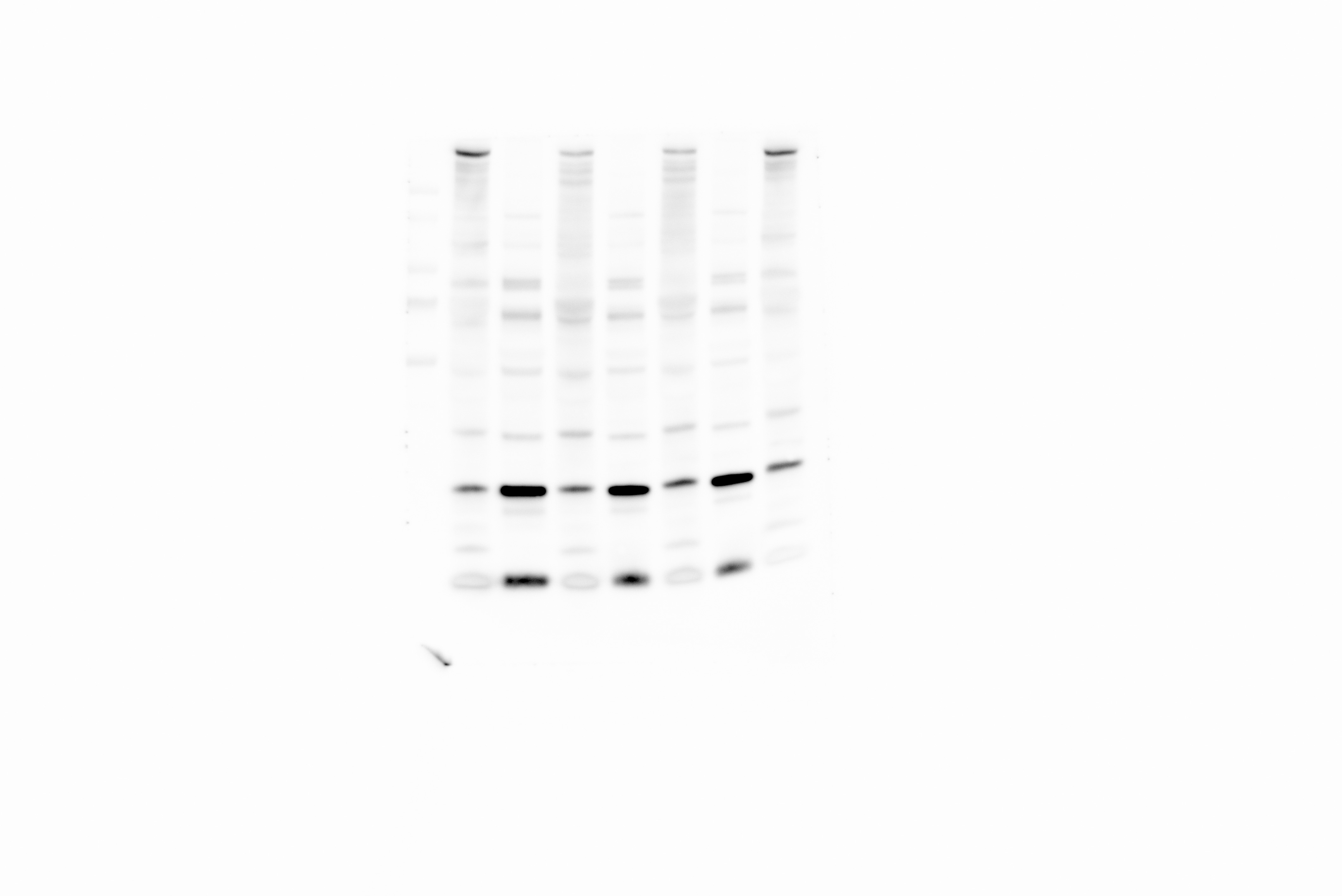

Supplement: Supplementary file 1 [file DataSheet1.zip › original image files for WB/Lung/tmem-lung/TMEM.jpg]

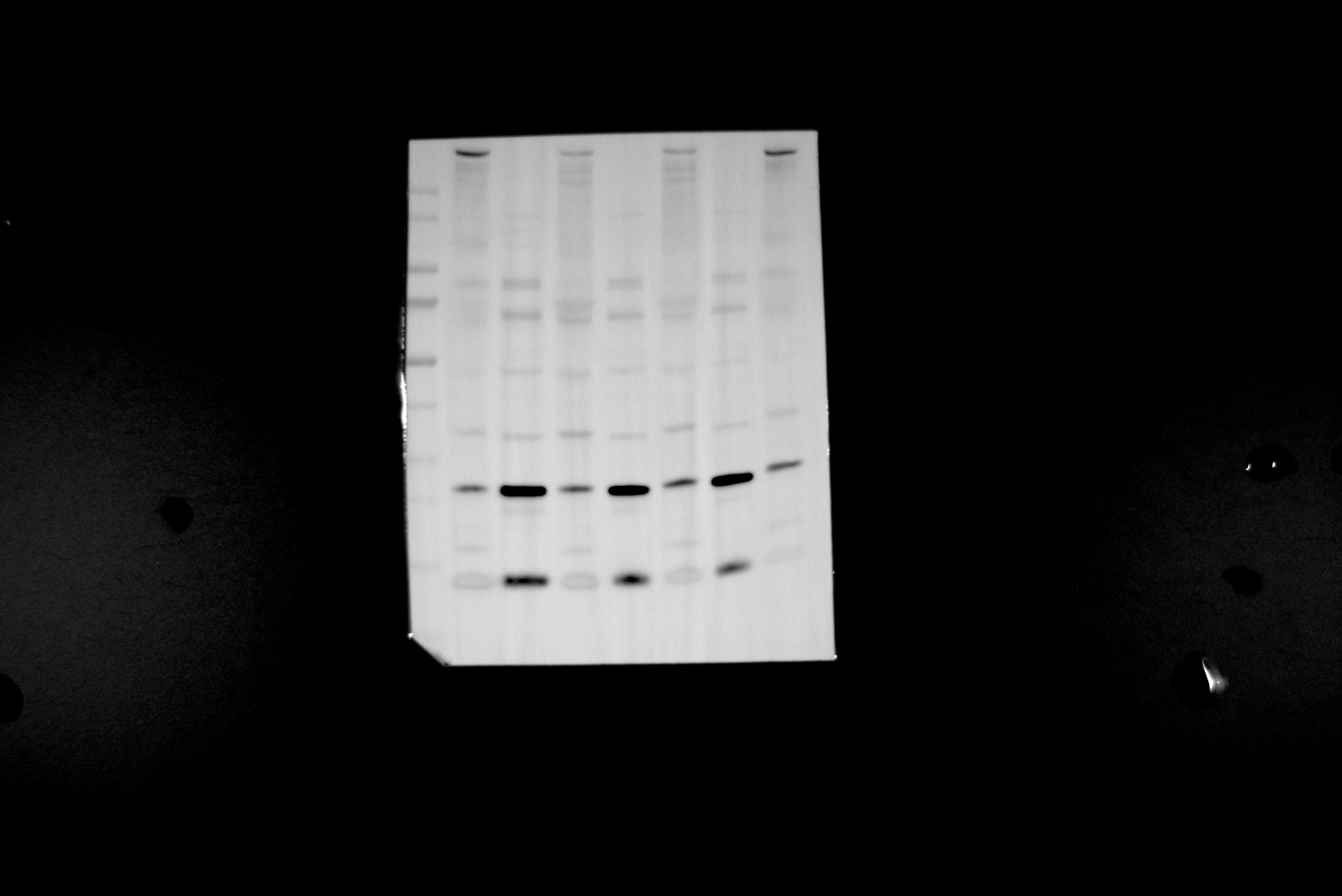

Supplement: Supplementary file 1 [file DataSheet1.zip › original image files for WB/Lung/tmem-lung/TMEM1.jpg]

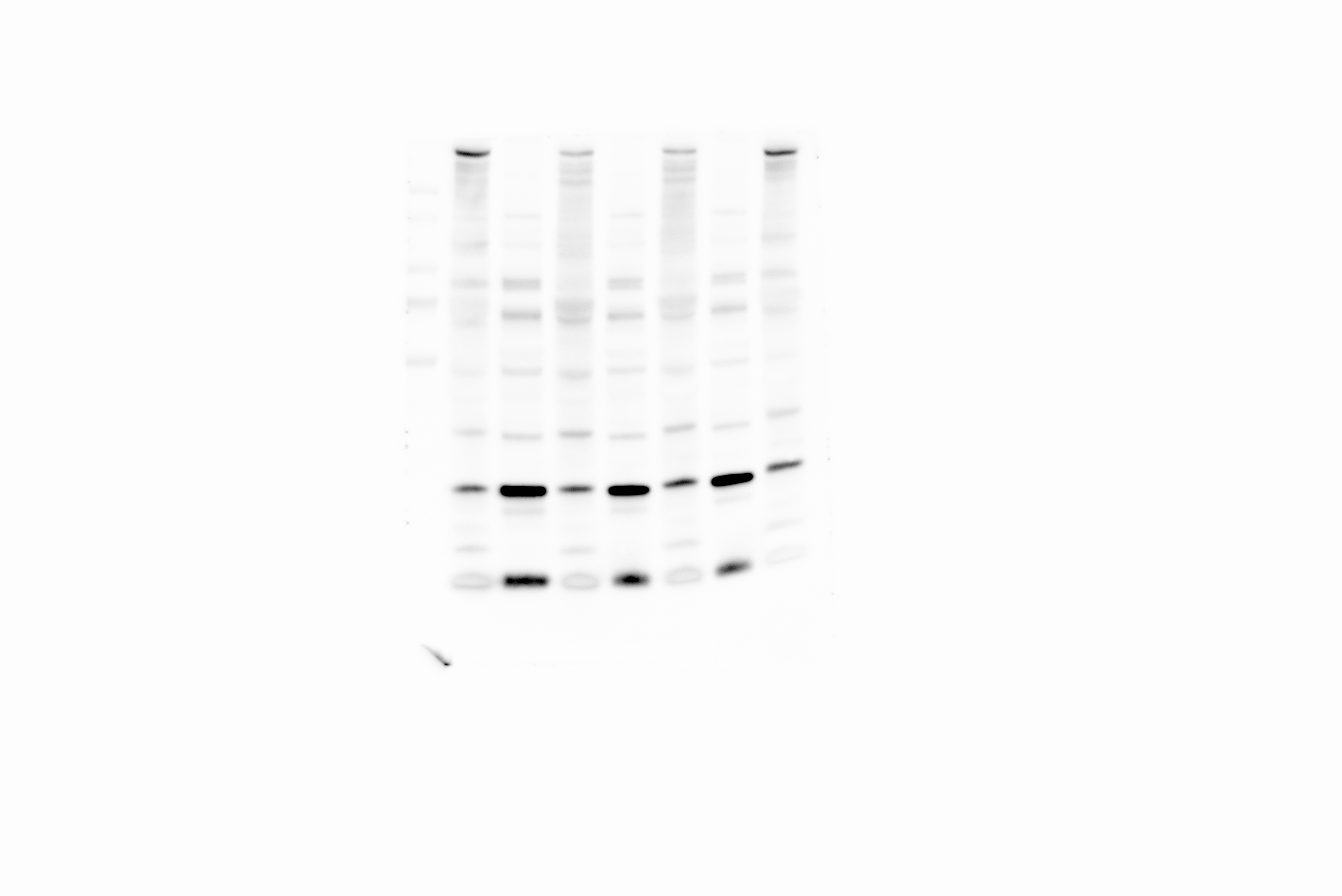

Supplement: Supplementary file 1 [file DataSheet1.zip › original image files for WB/Lung/tmem-lung/TMEM2.jpg]

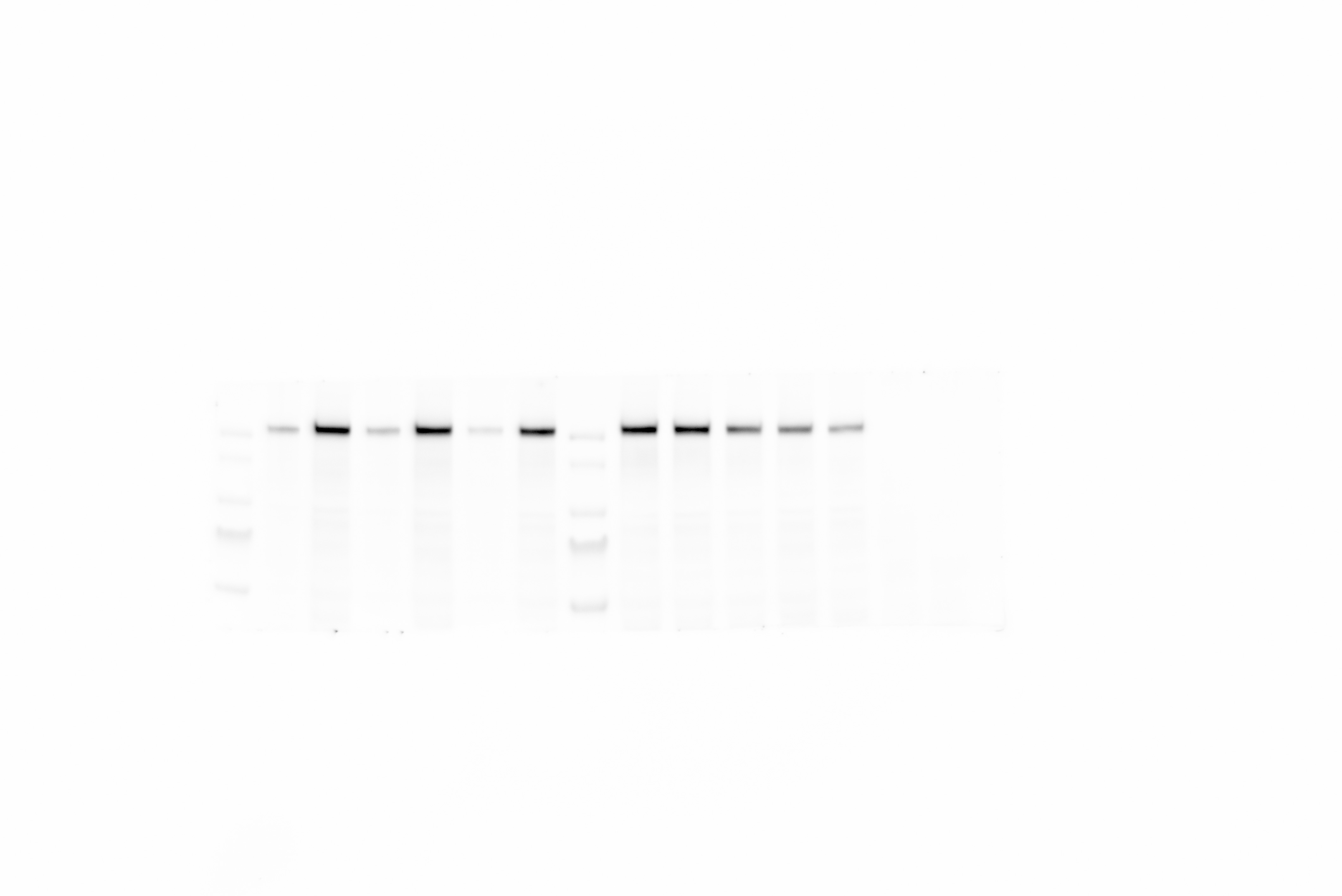

Supplement: Supplementary file 1 [file DataSheet1.zip › original image files for WB/Pancreas/CADPS &YZ/CAPS1.jpg]

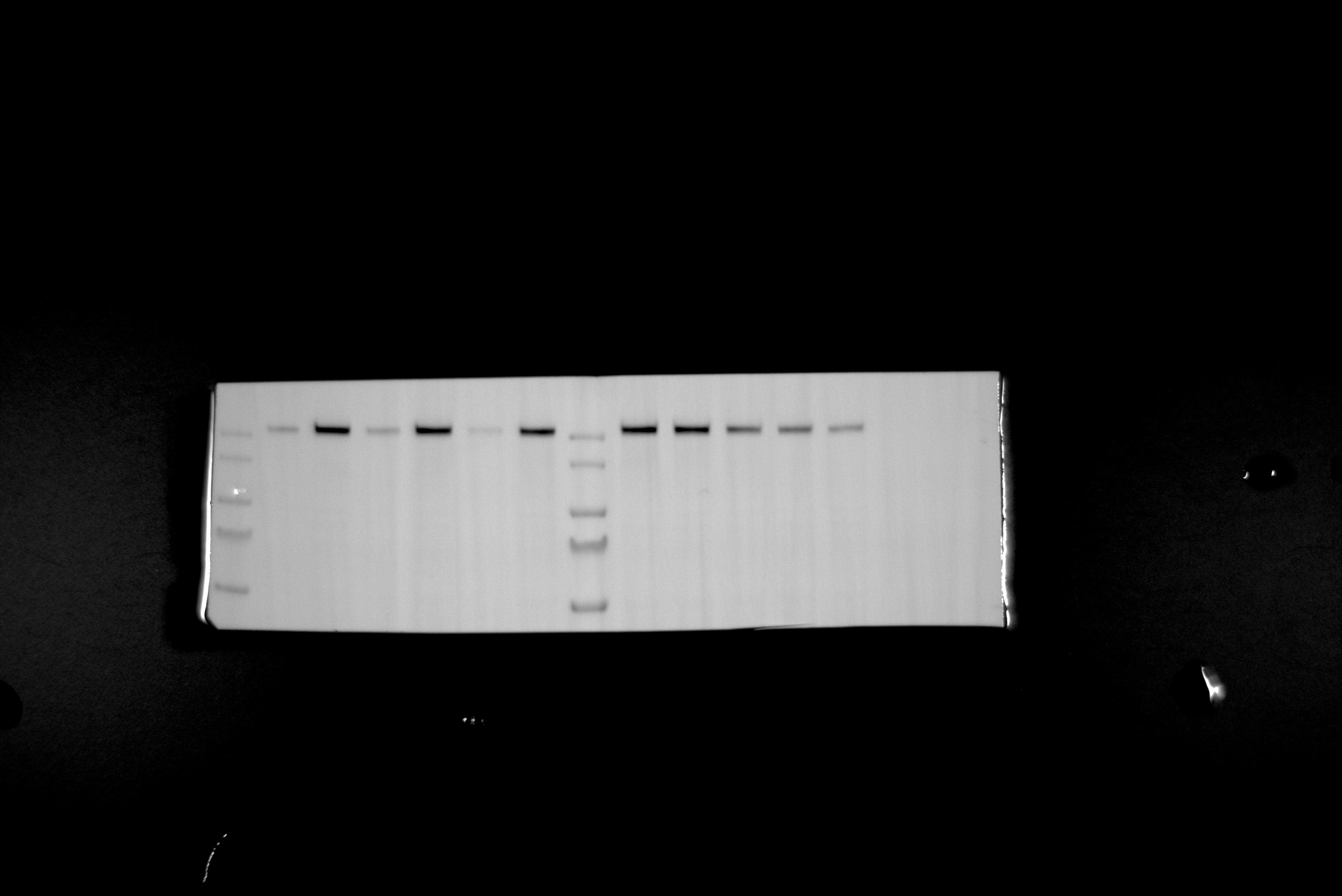

Supplement: Supplementary file 1 [file DataSheet1.zip › original image files for WB/Pancreas/CADPS &YZ/CAPS12.jpg]

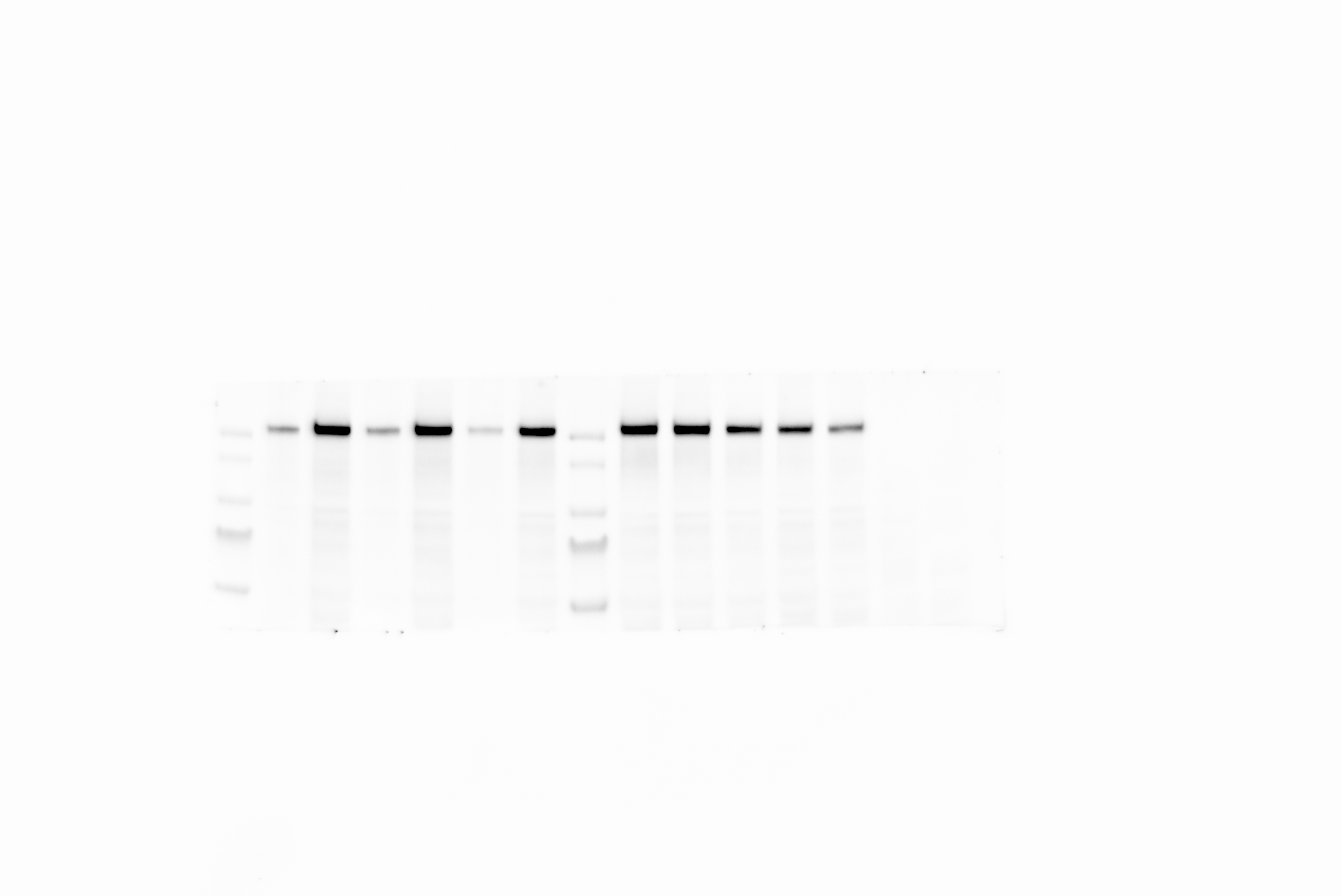

Supplement: Supplementary file 1 [file DataSheet1.zip › original image files for WB/Pancreas/CADPS &YZ/CAPS13.jpg]

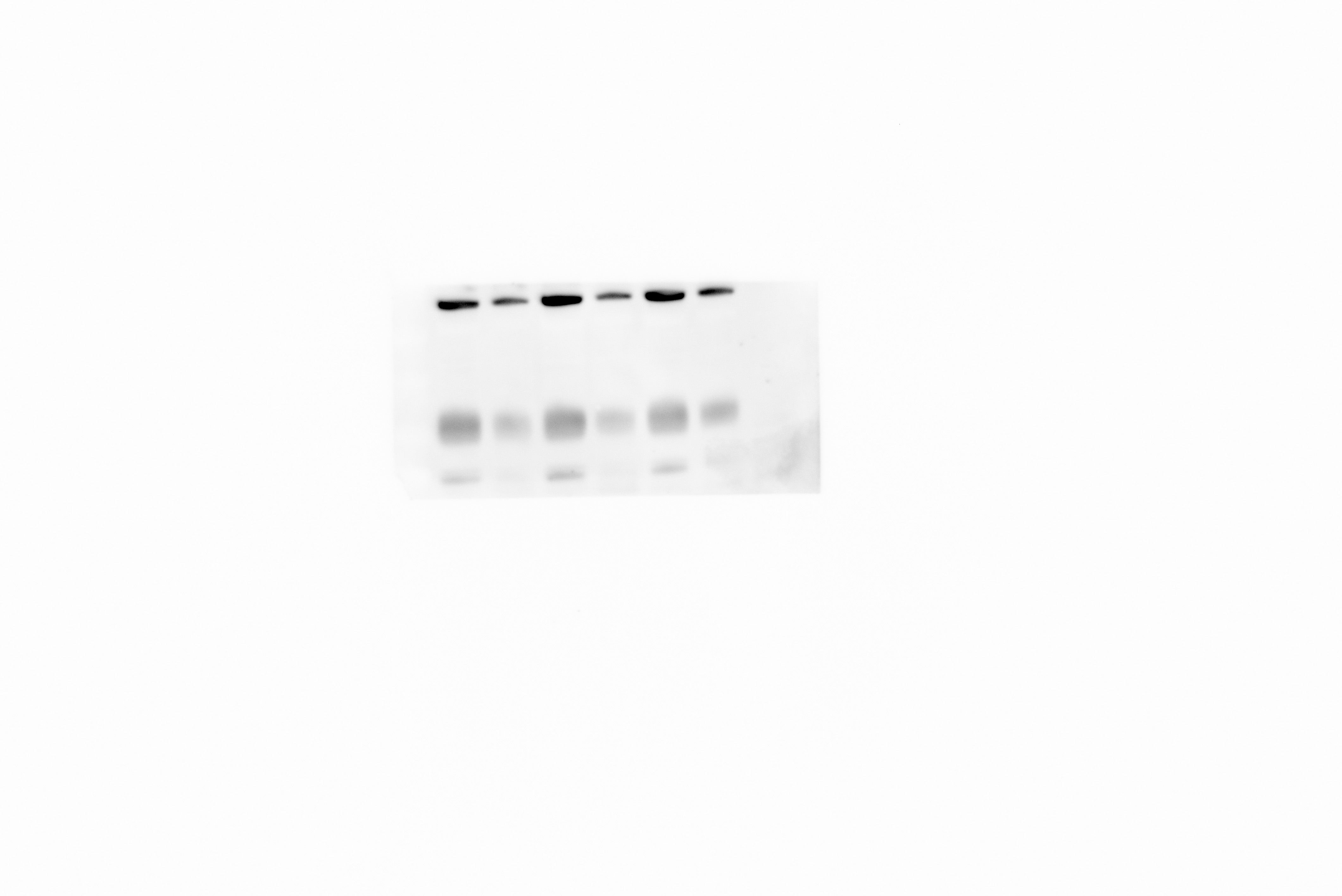

Supplement: Supplementary file 1 [file DataSheet1.zip › original image files for WB/Pancreas/EDNRB &YZ/EDNRB.jpg]

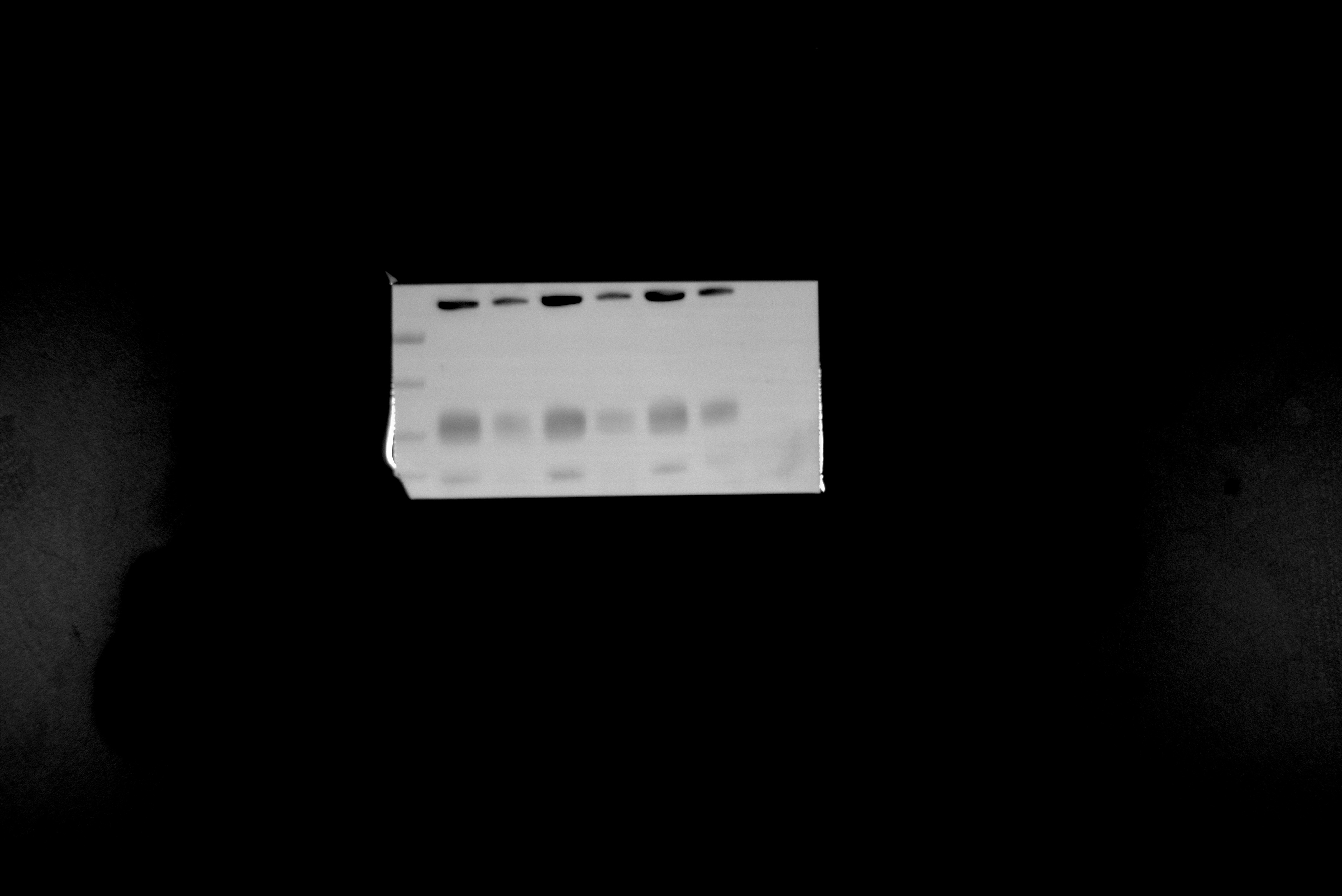

Supplement: Supplementary file 1 [file DataSheet1.zip › original image files for WB/Pancreas/EDNRB &YZ/EDNRB-1.jpg]

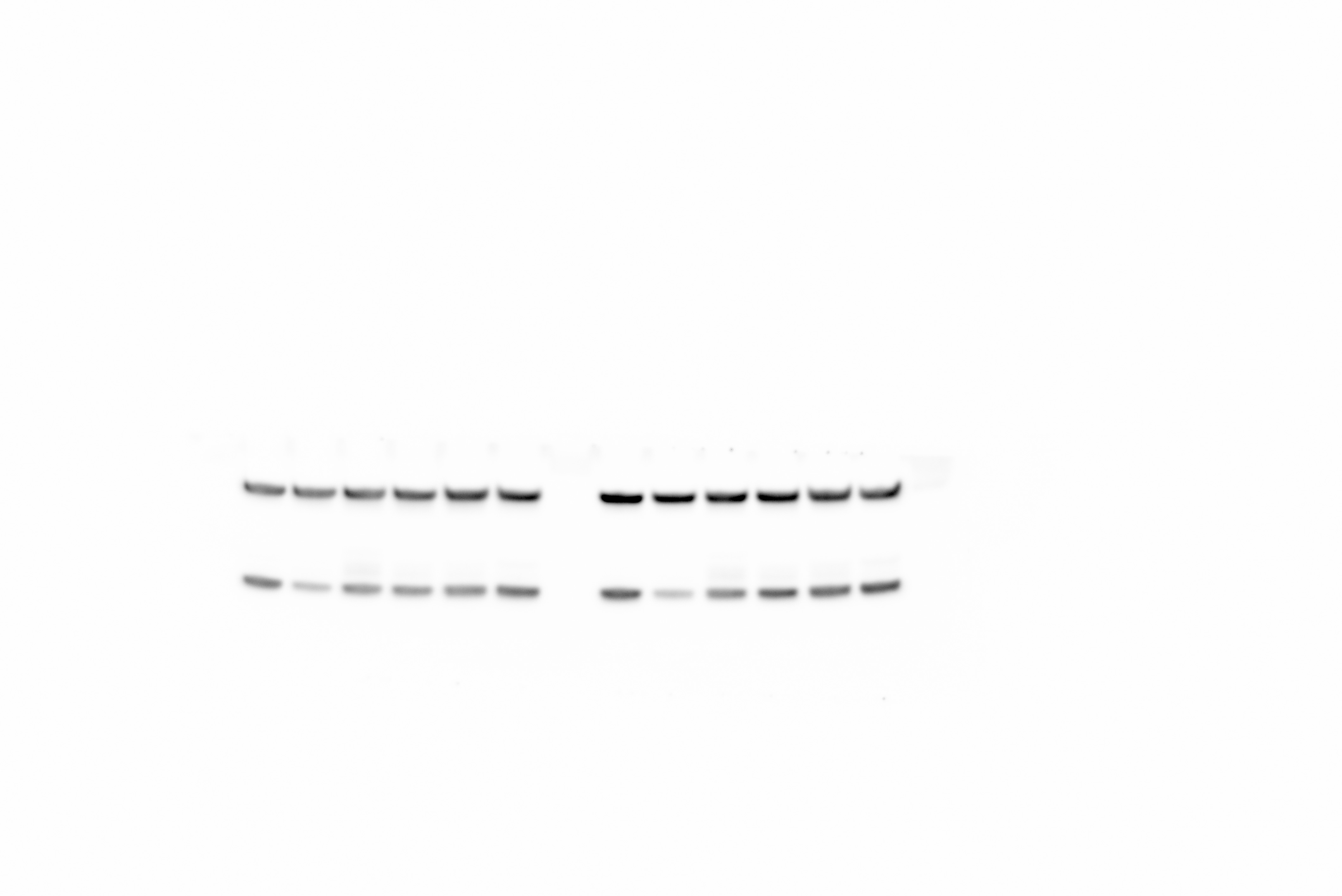

Supplement: Supplementary file 1 [file DataSheet1.zip › original image files for WB/Pancreas/GAPDH& YZ/GAPDH.jpg]

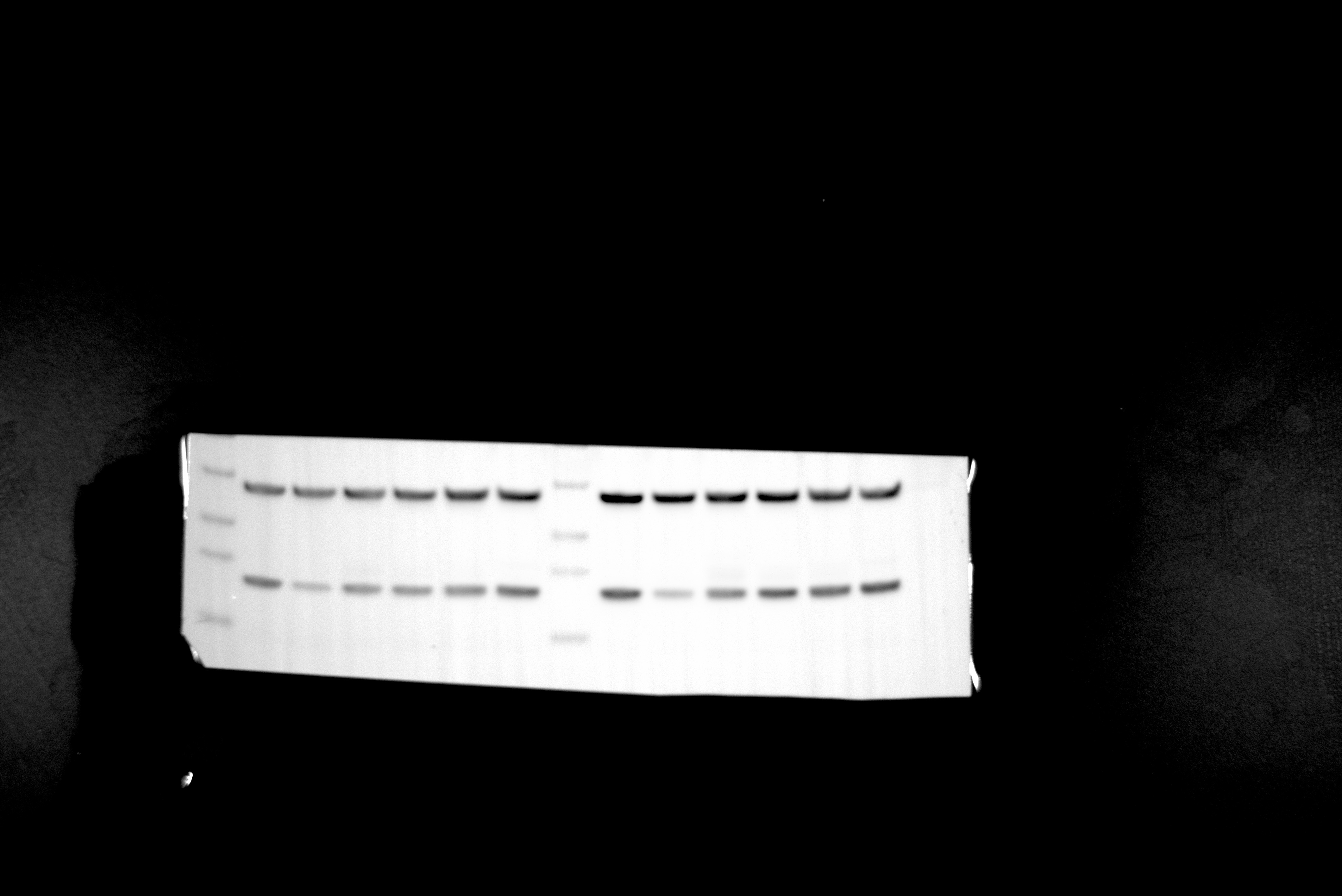

Supplement: Supplementary file 1 [file DataSheet1.zip › original image files for WB/Pancreas/GAPDH& YZ/GAPDH-1.jpg]

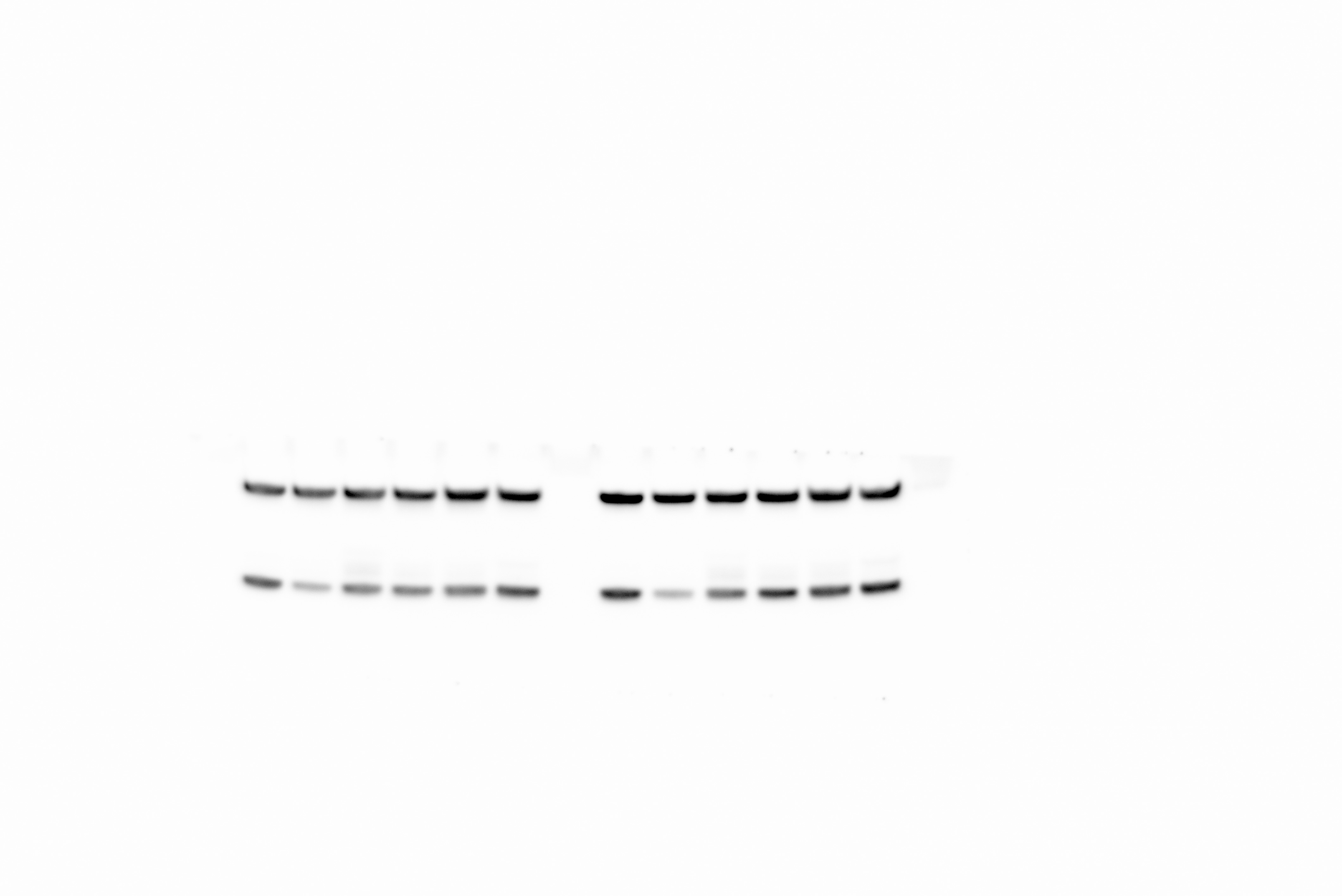

Supplement: Supplementary file 1 [file DataSheet1.zip › original image files for WB/Pancreas/GAPDH& YZ/GAPDH-2.jpg]

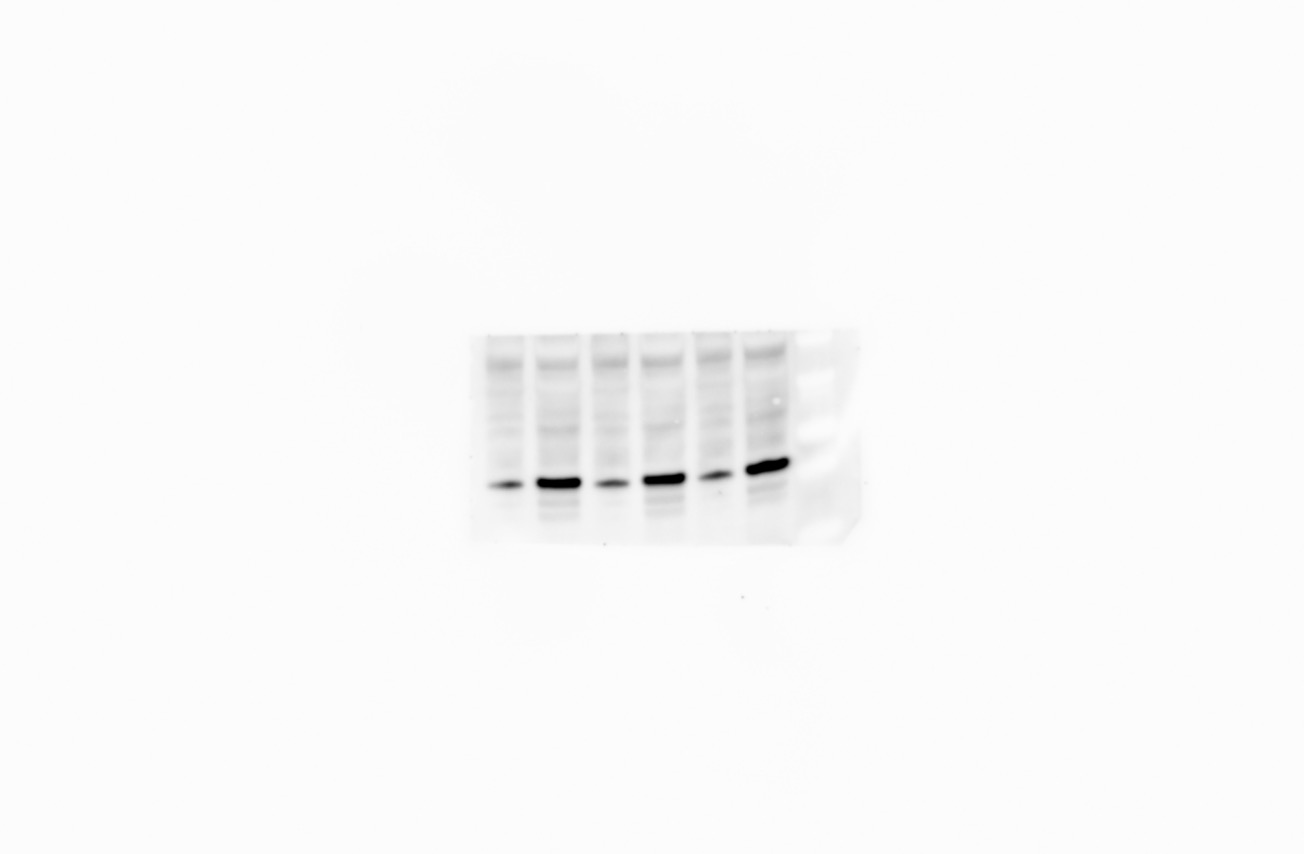

Supplement: Supplementary file 1 [file DataSheet1.zip › original image files for WB/Pancreas/TMEM1 &YZ/TMEM.jpg]

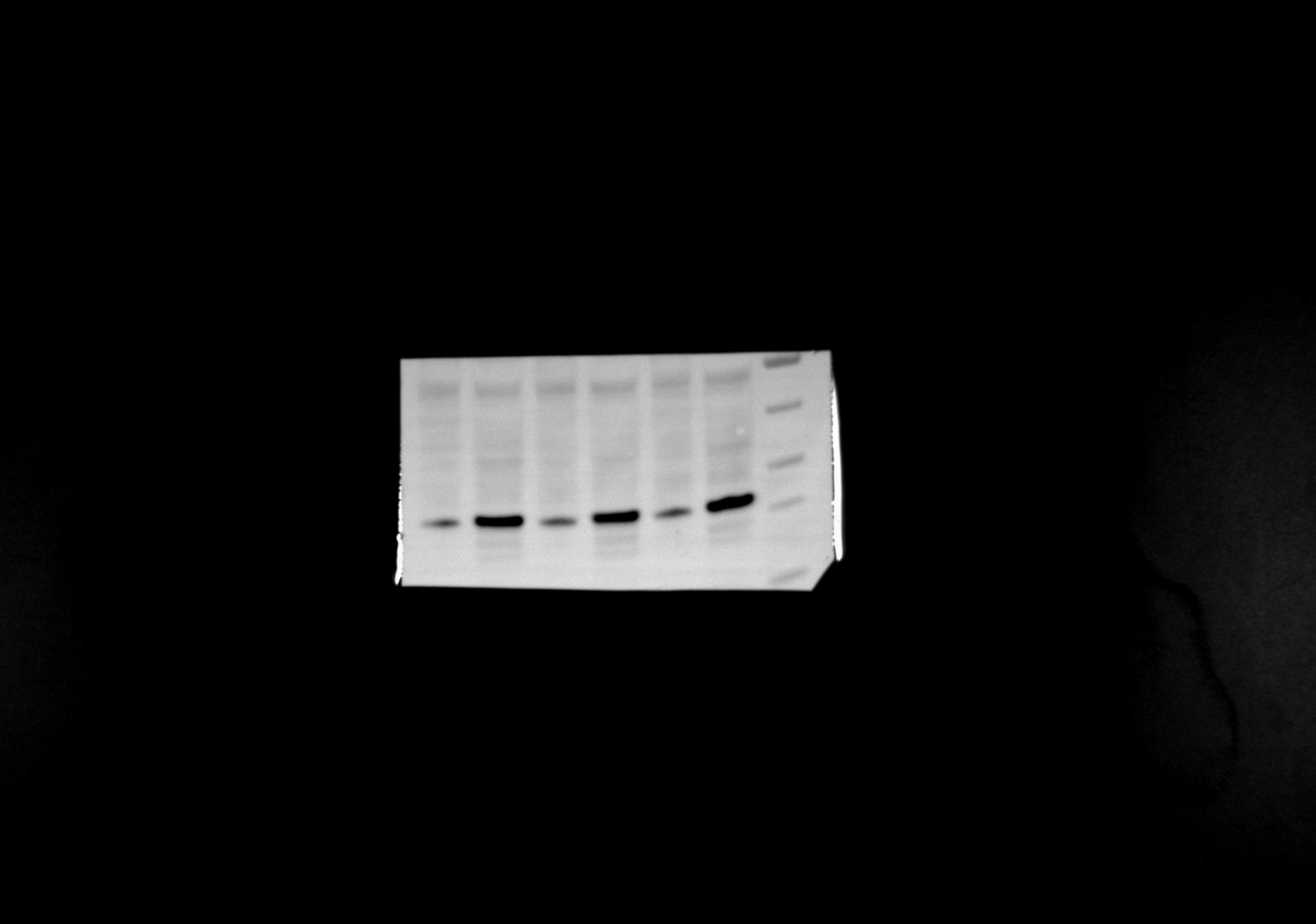

Supplement: Supplementary file 1 [file DataSheet1.zip › original image files for WB/Pancreas/TMEM1 &YZ/TMEM2.jpg]
